# The Polymorphisms of lncRNA HOXA11-AS and the risk of Lung Cancer in Northeastern Chinese population

**DOI:** 10.7150/jca.35411

**Published:** 2020-01-01

**Authors:** Min Gao, Hang Li, Yanhong Bi, Ziwei Zhang, Shengli Wang, Juan Li, Zitai Yang, Xiaoting Lv, Baosen Zhou, Zhihua Yin

**Affiliations:** 1 Department of Epidemiology, School of Public Health, China Medical University, Shenyang 110122, PR China; 2 Key Laboratory of Cancer Etiology and Intervention, University of Liaoning Province, Shenyang 110122, PR China

**Keywords:** Single nucleotide polymorphisms, Lung cancer, LncRNA, HOXA11-AS, Interaction.

## Abstract

Long non-coding RNAs (LncRNA) have been wildly explored in several malignant tumors. This study aimed to evaluate the effect of HOXA11-AS polymorphisms (rs17427875 and rs11564004) on lung cancer susceptibility and its interaction with smoking exposure. This hospital-based case-control study, which included 466 cases and 557 controls, was carried out in Shenyang City, Liaoning province. The genotyping method was TaqMan allelic discrimination assay and all statistical analysis were performed by SPSS 20.0 and R (3.5.3). The results demonstrated that HOXA11AS-rs17427875 polymorphisms were correlated with the susceptibility of lung adenocarcinoma. T alleles of rs17427875 played a portal role in increasing lung adenocarcinoma risk. HOXA11AS-rs11564004 polymorphisms had the significant association with lung cancer risks, as well as its subtypes like non-small cell lung cancer, adenocarcinoma. The allele G of rs11564004 acted as a protective factor for lung cancer. The similar results were observed in the homozygous model and recessive model of rs11564004. Nevertheless, interaction analysis of the additive and multiplicative model scales showed no statistical significance between HOXA11-AS polymorphisms and smoking exposure in the development of lung cancer.

## Background

Malignant tumors are major public health problems. Cancer constitutes an enormous burden on society in developing and developed countries 1. Lung cancer is a kind of a malignant tumor with high incidence and mortality. An estimated 1.8 million new lung carcinoma cases can be diagnosed annually, which was the most frequently diagnosed cancer type 2. It is also the leading cause of cancer death in male and the second in female worldwide^3^. According to World Health Organization (WHO), lung cancer can (LC) be divided into non-small cell lung cancer (NSCLC) and small cell lung cancer (SCLC), while NSCLC accounts for 85%. NSCLC can be further classified into three subtypes: lung adenocarcinoma (LUAD), lung squamous cell carcinomas (LUSC) and small cell carcinomas (SCC).

In China, lung cancer is the leading cause of death, resulted in 25.04% of cancer-related deaths, accounting for 19.3% in total diagnosed cancer 4, 5. Smoking is currently considered to be the most important environmental risk factor of lung cancer, but statistical research showed that 15-25% of lung cancer patients have no history of tobacco exposure. The proportion of female cases without prominent tobacco exposure was as high as 53 %^6^, which indicated that smoking exposure was not the essential factor in the development of lung cancer. Gene mutations, environmental factors and their interactions have been extensively studied in recent years. With the in-depth exploration of genome-wide association studies (GWAS), a variety of studies have emerged for genetic risk factors, which have a potential association between single nucleotide polymorphisms (SNPs) in LncRNA and various cancer types.

Long non-coding RNA (LncRNA), greater than 200 nt with no transcription protein, is mainly produced by RNA polymerase II transcription, which lacking of transcription for encoding protein and functional opening-read frame^7^, ^8^. LncRNA is involved in a variety of biological process, including molecular genetics, cell differentiation and cancer cell progression^9^-^11^, such as H19 in breast cancer, HOTAIR in lung cancer, etc 12, 13.

The HOXA is a transcription factor of homeobox gene family. HOXA11-AS (also named HOXA11S, HOXA-AS5, HOXA11AS, HOXA11-AS1 and NCRNA00076), is a kind of LncRNA transcribed from the antisense strand of HOXA11 gene, located on chromosome 7p15.2. The ectopic expression of HOXA11-AS plays a crucial role in the process of several cancer types, like expressing with a low level in colorectal cancer and high level in breast cancer, non-small cell lung cancer, etc^14^-^16^. At present, whether gene mutation caused the ectopic expression of HOXA11-AS has not been clearly elucidated. Few reports had explored SNPs on HOXA11-AS sequence with lung cancer susceptibility. Only Edward J et al. studied the association between HOXA11AS-rs17427875 and serous ovarian cancer (EOC) by GWAS^17^. The relationship between HOXA11-AS SNPs and lung cancer is still worthy of further exploration.

## Materials and Methods

### Analysis of HOXA11-AS expression in TCGA database

The RNA-seq data profiles and prognosis information of LUAD and LUSC tissues were downloaded from The Cancer Genome Atlas (TCGA) databases before 20^th^ March, 2019 (https://portal.gdc.cancer.gov/), including 54 normal/497 LUAD tissues, 49 normal/502 LUSC tissues. Data extraction and integration were utilized by Perl (v5.24.3) and the clinical expression was analyzed by the “edger” “survival” R package (v3.5.3). *In silico analysis* was conducted by SNPinfo web server (https://manticore.niehs.nih.gov/) and RegulomeDB (http://regulomedb.org/).

### Participants including/excluding criteria

The hospital-based study was carried out in Shenyang, Liaoning Province. 466 cases and 557 controls were included in this study. All participants were enrolled from affiliated hospitals of China Medical University, while all controls were cancer-free population. The criteria of included cases are listed as follows: (a) Patients who have been confirmed by histopathology and newly diagnosed lung cancer; (b) Patients have no previous cancer; (c) Patients have no received radiation or chemotherapy history before. The criteria of included controls are consistent with (b) (c) description. A few controls have been diagnosed as no cancer disease, like hypertension, angina pectoris and coronary heart disease, etc. All enrolled controls came from medical examination centers of the affiliated hospitals in the same period, matched to cases with age about ±5 years. The Institutional Review Board of China Medical University agreed to this investigation. All participants had the right to know about the research and had voluntarily signed informed consent. We defined less than 100 cigarettes smoking in a lifetime as non-smokers.

### Genotyping Methods

Genomic DNA samples were isolated from venous blood by using the phenol-chloroform method. 10 mL of venous blood was obtained from every enrolled volunteer. TaqMan allelic discrimination assay (Applied Biosystems, Foster City, CA, USA) was used to genotyping by using 7500 fast real-time PCR system (Applied Biosystems, Foster City, CA, USA).

### Statistical Analysis

We used Pearson chi-square test (categorical variables) and Student's t-test (continuous variables) to calculate the distribution difference between cases and controls. Unconditional logistic regression was utilized to calculate joint ORs and adjusted ORs, 95% confidence interval (CIs). Cumulative effects were calculated by linear-by-linear association of χ2 test. Additive and multiplicative interaction were analyzed to embody gene-environment interaction. All tests were two-sided and p<0.05 was regarded as having statistical significance. The above calculations were obtained from SPSS20.0 (IBM SPSS, Inc. Chicago, IL, USA).

## Results

### Expression analysis of HOXA11-AS in LUAD and LUSC tissues of Lung Cancer

**Figure 1:**
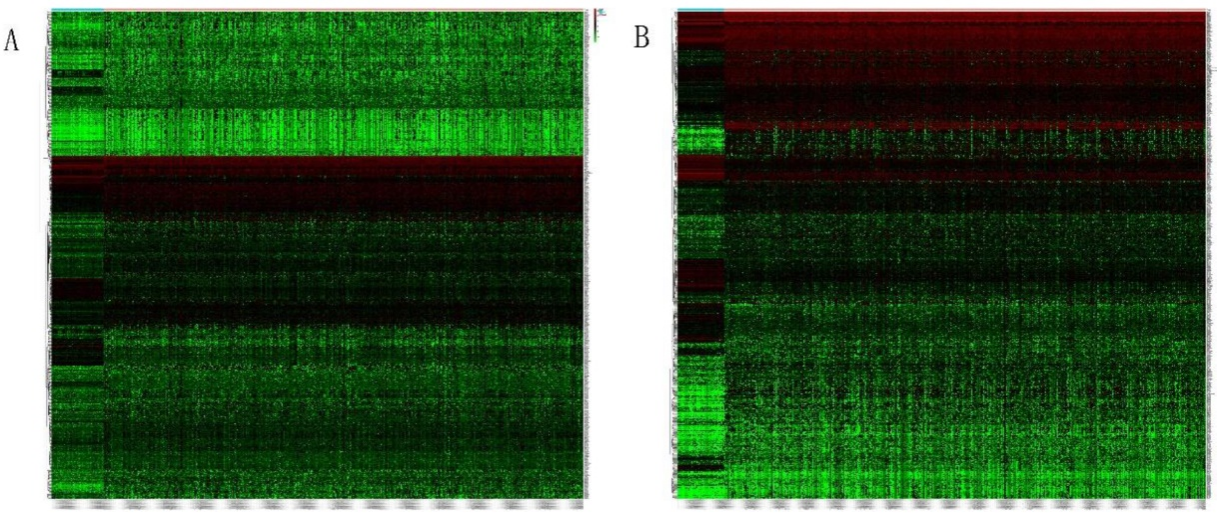
Heatmap of differentially expressed LncRNAs in LUAD and LUSC tissues. (A: LUAD tissues; B: LUSC tissues).

**Figure 2:**
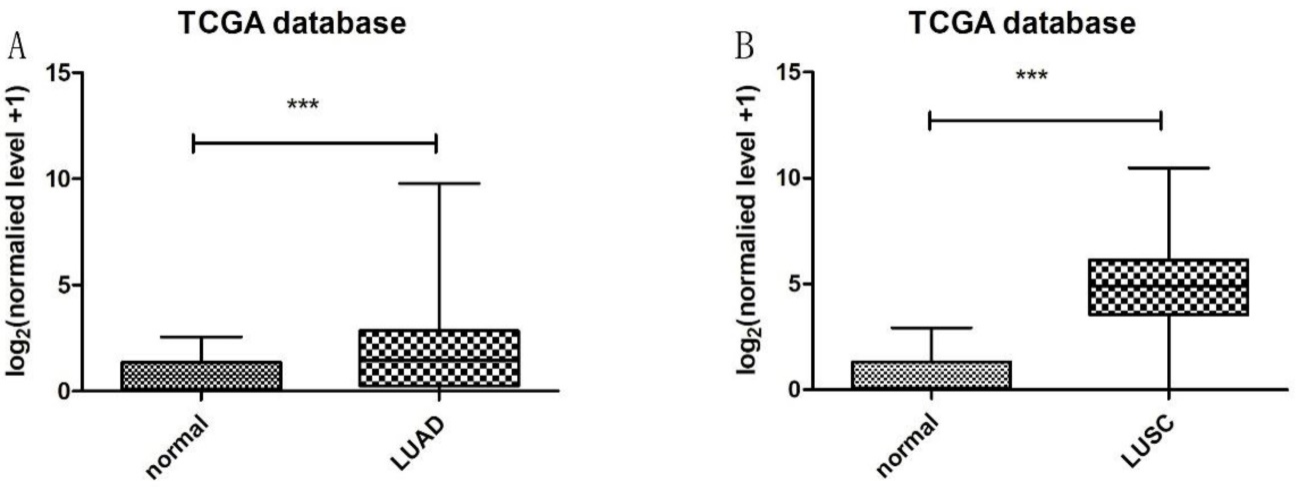
Expression of LncRNA HOXA11-AS in LUAD and LUSC tissues samples from TCGA database. (A: normal=54, LUAD=497, P<0.001; B: normal=49, LUSC=502, P<0.001).

**Figure 3:**
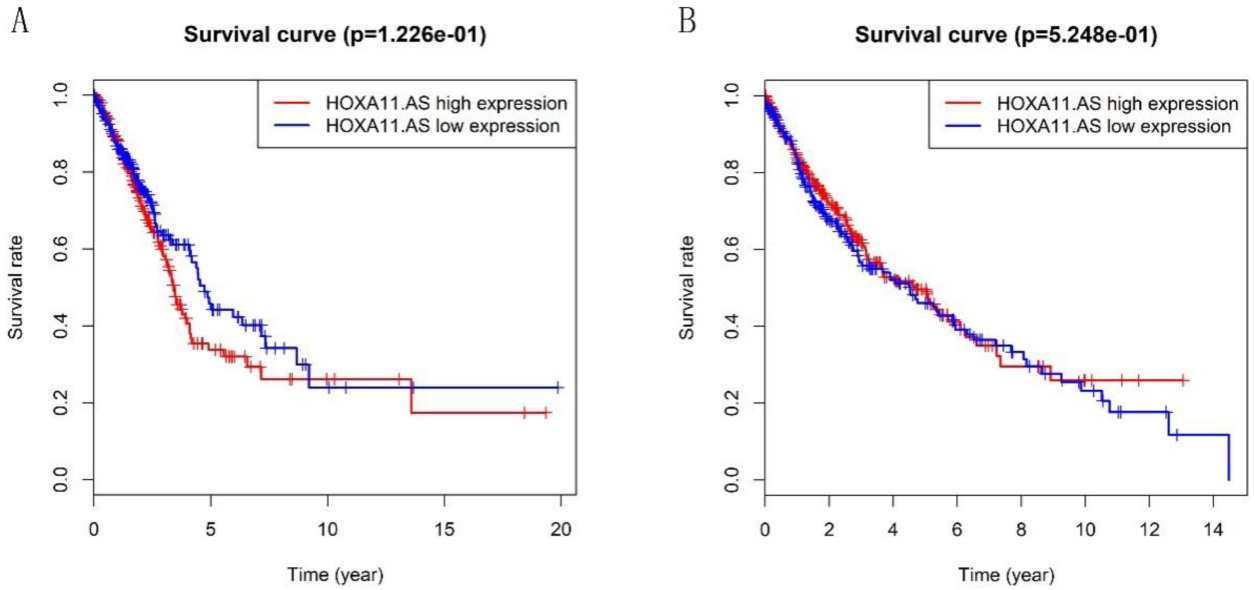
Validation of the prognostic lncRNA HOXA11-AS in TCGA database. (A: Kaplan‐Meier curve of the risk score for the OS of LUAD patients in TCGA, P=0.122; B: Kaplan‐Meier curve of the risk score for the OS of LUSC patients in TCGA, P=0.525).

Heatmap in Figure 1A and 1B showed massive LncRNA differentially expressed in LUAD and LUSC tissues, and the expression level of LncRNA HOXA11-AS was significantly elevated in lung cancer tissues (logFC=4.337, logCPM=-1.286, P<0.001; logFC=6.017, logCPM= 0.362, P<0.001) (Supplementary table 1, Supplementary table 2). Besides, the expression of LUAD and LUSC tissues were significantly higher than that in normal tissues through analyzing the data from TCGA database by edger.R function (Figure 2A and 2B, P<0.001). The overall survival time gathered from in TCGA clinical data showed negative results between clinical samples and the expression level of LncRNA HOXA11-AS by survival. R function analysis (Figure 3A P=0.123, Figure 3B P=0.525).

### Relative Association Analysis of HOXA11-AS and Lung Cancer Susceptibility

As shown in Table 1, 466 cases (241 males, 225 females) and 557 controls (291 males, 266 females) were enrolled in this study. Among the cases, there were 155 Squamous cell carcinomas (LUSC), 241 Adenocarcinomas (LUAD), and 61 Small cell carcinomas (SCC). The mean and standard deviation (Mean ± SD) of cases and controls were 59.66±10.765 and 59.06 ±14.386, respectively. The distribution of sample ages had no statistical significance examining by Student's t-test(P=0.441). However, the individuals with smoking history had remarkable differences in cases and controls (P=0.001, OR=2.709). The genotyping distribution of controls were conformed to Hardy-Weinberg equilibrium (rs17427875= 0.355, rs11564004=0.430), indicating that the control group came from the same Mendelian genetic population.

The genotyping details of the SNPs were listed in Table 2 and Table 3, the allelic mutation of rs17427875 showed remarkable significance in lung adenocarcinoma population. As shown in Table 3, mutant allele T played a risky role in LUAD susceptibility (adjusted OR=1.937, P=0.005). Besides, compared to individuals with homozygous AA genotype, heterozygote AT genotype carriers could significantly increase the susceptibility of LUAD (adjusted OR=1.821, P=0.019). The dominant model also showed relationship between SNPs and the risk of LUAD (adjusted OR=1.883, P=0.013). As for rs11564004, the allelic mutation showed no statistical significance in lung cancer. However, the homozygous mutation allele GG carriers could notably decrease the risk of lung cancer comparing with TT genotype carriers (adjusted OR=0.505, P=0.024). The recessive model also showed significant results by comparing with GT+TT genotype carriers (adjusted OR=0.515, P=0.025). In NSCLC subtype analysis, both homozygous and recessive model presented a lessen effect in NSCLC susceptibility (adjusted OR=0.509, P=0.030; adjusted OR=0.531, P=0.039). According to the statistical results, we preliminarily indicated that the mutant allele G of rs11564004 may act as a protective factor in the risk of lung cancer.

To validate the possible function of tagSNP HOXA11AS-rs17427875 in cancers, two *in silico analysis* were utilized to predict the potential functions. A “3a” rank was got in RegulomeDB, which meant that HOXA11-AS rs17427875 SNP may be likely to affect binding transcription factors, motifs or DNase peak^18^. SNPinfo also predicted rs17427875 acting as a binding site for several transcription factors^19^.

Next, we analyzed cumulative effects of variant alleles rs17427875-T, rs11564004-T on lung cancer and its subtypes susceptibility (Table 4 and Table 5). The results elucidated that the increasing number of rs17427875-T, rs11564004-T and lung cancer susceptibility existed linear relationship, acting similarly with dose-dependent manner (LC: P=0.033, NSCLC: P=0.015, LUAD: P=0.025).

The crossover interaction analysis between SNPs and smoking exposure were listed in Table 6. We suggested that AA genotype carriers and AT+TT genotype carriers with smoking exposure were significantly connected with lung cancer (rs17427875: P=0.001; rs11564004: P=0.001). However, the parameters of additive and multiplicative interaction model presented that rs17427875 and rs11564004 polymorphisms had no remarkable interaction results with smoking exposure (showed in Table 7 and Table 8).

## Discussion

The biological function of HOXA11-AS in malignant tumors has been extensively explored in breast cancer, glioma, colorectal cancer, etc^14^, ^16^, ^20^. Moreover, depending on the cancer types and the environmental status of cancer cells, HOXA11-AS may act as both a proto-oncogene or a tumor suppressor gene. For instance, Li et al. demonstrated the lower expression level of HOXA11-AS in the colorectal cancer tissues and cell lines, which also correlated with tumor size, TNM stage and lymph node metastasis^14^. Nevertheless, the expression of HOXA11-AS in gastric cancer was higher than that in normal tissues, and this alternation affecting cell growth, migration, invasion, and apoptosis^21^.

The HOXA11-AS has been implicated in lung cancer but its biological mechanisms are still undocumented. The clinical data onto TCGA database elucidated that LncRNA HOXA11-AS expression was significantly increased in LUAD and LUSC of lung cancer compared with adjacent normal tissues. For now, four pathways were as follows: first, the higher expression levels of HOXA11-AS in NSCLC tissues predicted a poor prognosis. Knockdown HOXA11-AS in cancer cells dramatically inhibited cell invasive abilities, and meanwhile the transcription and protein levels of EMT related factors were down-regulated, but E-cadherin were increased in cells. The mechanistic results demonstrated that HOXA11-AS recruited EZH2 and DNMT1 to miR-200b promoter regions to restrain miR-200b expression in NSCLC cells^15^. Second, Yu et al. explored the functional association between LncRNA HOXA11-AS/miR-124/Sp1 axis. The study indicated that HOXA11-AS expression was negatively correlated with miR-124 in NSCLC tissues, but positively regulated target gene Sp1^22^. Third, comprehensive bioinformatics analysis were performed to explore the potential functional pathways in HOXA11-AS/miR-642b-3p/PDE4D^23^. Another analysis also speculated the HOXA11-AS function in NSCLC development, progression by regulating several pathways and genes, especially DOCK8 and TGF-β 24. At last, Zhao et al. demonstrated that HOXA11-AS played as a ceRNA to affect human lung adenocarcinoma development and chemoresistance via miR-454-3p/Stat3 axis^25^.

Edward et al. elucidated that HOXA11AS rs17427875-T was marginally associated with reduced EOC susceptibility in European ancestry population and the inhibitory effect was enhanced by alleles T^17^. Our study explored the association between HOXA11-AS polymorphisms and the risk of lung cancer in Northeastern Chinese population. The results demonstrated that rs17427875-T, rs11564004-T were correlated with the increasing lung cancer risks. Binding* in silico analysis*, it can be inferred that SNPs may inhibit or promote the occurrence and development of lung cancer by associating with the transcription factors related to lung cancer. And in the light of the cumulative effects, the risk of lung cancer was increased with the growing number of dangerous alleles. While, no additive or multiplicative interaction were obtained between gene polymorphisms and smoking status.

Several limitations can be emphatically highlighted from this study. First, the participants from Shenyang city hardly represent all northeastern Chinese population; second, the sample size is not large enough to make it possible for the results to be false-positive or false-negative; Third, no functional verification of these SNPs was carried out in this study. State thus, a larger size population and biological mechanism research still need to be further explored.

## Conclusion

HOXA11AS-rs17427875 and rs11564004 were correlated with the risk of lung cancer as well as its subtypes. And meanwhile, there were dose-response effect between the increasing number of rs1727875-T and rs11564004-T with lung cancer procedure. No additive or multiplicative interaction with smoking status could be gain.

## Supplementary Material

Supplementary table 1.

Supplementary table 2.

## Figures and Tables

**Table 1:** Basic characteristics including in this study.

	Case(n=466)	Control(n=557)	P
**Gender**			0.866
male	241(51.7%)	291(52.2%)	
female	225(48.3%)	266(47.8%)	
**Age (years)**			0.072
≤59	216(46.4%)	227(40.8%)	
>59	250(53.6%)	330(59.2%)	
Mean ± SD	59.66 ±10.765	59.06 ±14.386	0.441
**Histology**			
Squamous cell carcinoma	155(33.3%)		
Adenocarcinoma	241(51.7%)		
Small cell carcinoma	61(13.1%)		
Other cancer types	9(1.9%)		
**Smoking exposure**			
Yes	236(50.6%)	153(27.5%)	0.001
No	230(49.4%)	404(72.5%)	

**Table 2:** Association between the two SNPs and risk of lung cancer and non-small cell lung cancer.

	No. of controls(%)	Lung Cancer			Non-small cell lung cancer		
		No.(%)	OR(95%CI)^a^	P	No.(%)	OR(95%CI)^a^	P
rs17427875							
AA	515(92.5)	419(89.9)	1(ref.)		361(89.1)	1(ref.)	
AT	42(7.5)	45(9.7)	1.365(0.862-2.161)	0.184	42(10.4)	1.464(0.918-2.334)	0.110
TT	0	2(0.4)	-	-	2(0.5)	-	-
AT+TTvsAA			1.423(0.903-2.244)	0.128		1.531(0.965-2.429)	0.071
TTvsAT+AA			-	-		-	-
A allele	1072	883	1(ref.)		764	1(ref.)	
T allele	42	49	1.416(0.929-2.159)	0.104	46	1.537 (1.001-2.359)	0.048
rs11564004							
TT	312(56.0)	274(58.8)	1(ref.)		242(59.8)	1(ref.)	
GT	205(36.8)	173(37.1)	0.952(0.725-1.250)	0.724	146(36.0)	0.896(0.676-1.188)	0.446
GG	40(7.2)	19(4.1)	0.505(0.280-0.912)	0.024	17(4.2)	0.509(0.276-0.938)	0.030
GT+GGvsTT			0.876(0.675-1.138)	0.322		0.831(0.634-1.089)	0.180
GGvsGT+TT			0.515(0.288-0.921)	0.025		0.531(0.291-0.969)	0.039
T allele	829	721	1(ref.)		630	1(ref.)	
G allele	285	211	1.175(0.958-1.441)	0.122	180	0.831(0.671-1.029)	0.089

a: adjusted by age, gender and smoking

**Table 3:** Association between the two SNPs and risk of lung adenocarcinoma and squamous cell lung cancer.

	No. of controls(%)	Lung Adenocarcinoma			Squamous cell carcinoma		
		No.(%)	OR(95%CI)^a^	P	No.(%)	OR(95%CI)^a^	P
rs17427875							
AA	515(92.5)	208(86.3)	1(ref.)		144(92.9)	1(ref.)	
AT	42(7.5)	32(13.3)	1.821(1.103-3.007)	0.019	10(6.5)	0.881(0.406-1.914)	0.749
TT	0	1(0.4)	-	-	1(0.6)	-	-
AT+TTvsAA			1.883(1.145-3.095)	0.013		0.996(0.470-2.110)	0.992
TTvsAT+AA			-	-		-	-
A allele	1072	448	1(ref.)		298	1(ref.)	
T allele	42	34	1.937(1.216-3.085)	0.005	12	1.028(0.534-1.977)	0.935
rs11564004							
TT	312(56.0)	142(58.9)	1(ref.)		97(62.6)	1(ref.)	
GT	205(36.8)	87(36.1)	0.922(0.664-1.281)	0.628	53(34.2)	0.822(0.544-1.241)	0.350
GG	40(7.2)	12(5.0)	0.632(0.318-1.257)	0.191	5(3.2)	0.366(0.133-1.006)	0.051
GT+GGvsTT			0.874(0.638-1.196)	0.400		0.743(0.500-1.106)	0.144
GGvsGT+TT			0.653(0.332-1.282)	0.216		0.395(0.145-1.071)	0.068
T allele	829	371	1(ref.)		247	1(ref.)	
G allele	285	111	0.870(0.677-1.119)	0.278	63	0.742(0.545-1.009)	0.057

a: adjusted by age, gender and smoking

**Table 4:** Cumulative effects of rs17427875-T and rs11564004-T on lung cancer and non-small cell lung cancer susceptibility.

	No. of controls(%)	Lung Cancer			Non-small cell lung cancer		
		No.(%)	OR(95%CI)^a^	P	No.(%)	OR(95%CI)^a^	P
No. of alleles							
0(ref.)	35(6.3)	18(3.9)	1(ref.)		16(4.0)	1(ref.)	
1	195(35.0)	152(32.6)	1.636(0.870-3.075)	0.126	127(31.4)	1.519(0.790—2.922)	0.210
2	305(54.8)	270(57.9)	1.867(1.009-3.455)	0.047	237(58.5)	1.846(0.977-3.489)	0.059
3-4	22(3.9)	26(5.6)	2.598(1.126-5.990)	0.025	25(6.2)	2.794(1.191-6.550)	0.018
Trend			P^b^ =0.033			P^b^ =0.015	-
0	35(6.3)	18(3.9)	1(ref.)		16(4.0)	1(ref.)	
1-4	522(93.7)	448(96.1)	1.811(0.988-3.317)	0.055	389(96.0)	1.761(0.942-3.295)	0.076

a: adjusted by age, gender and smoking. b: P value was calculated by linear-by-linear association of χ2 test.

**Table 5:** Cumulative effects of rs17427875-T and rs11564004-T on squamous cell carcinoma and adenocarcinoma susceptibility.

	No. of controls(%)	Squamous cell carcinoma			Lung Adenocarcinoma		
		No.(%)	OR(95%CI)^a^	P	No.(%)	OR(95%CI)^a^	P
No. of alleles							
0(ref.)	35(6.3)	5(3.2)	1(ref.)		11(4.6)	1(ref.)	
1	195(35.0)	48(31.0)	2.020(0.709-5.751)	0.188	73(30.3)	1.232(0.587-2.589)	0.581
2	305(54.8)	95(61.3)	2.531(0.912-7.025)	0.075	139(57.7)	1.506(0.733-3.094)	0.265
3-4	22(3.9)	7(4.5)	2.835(0.735-10.930)	0.130	18(7.5)	2.677(1.044-6.862)	0.040
Trend			P^b^=0.074			P^b^ =0.025	
0	35(6.3)	5(3.2)	1(ref.)		11(4.6)	1(ref.)	
1-4	522(93.7)	150(96.8)	2.352(0.858-6.451)	0.097	230(95.4)	1.453(0.716-2.948)	0.301

a: adjusted by age, gender and smoking. b: P value was calculated by linear-by-linear association of χ2 test.

**Table 6:** Crossover analysis of interaction between SNPs genotypes and smoking exposure in lung cancer.

	Genotype	Smoking	Controls (%)	Cases (%)	OR (95%CI)^a^	P
rs17427875	AA	Never	371(66.6)	201(43.1)	1(ref.)	
	AT+TT	Never	33(5.9)	29(6.2)	1.487(0.868-2.547)	0.148
	AA	Ever	144(25.9)	218(46.8)	5.403(3.718-7.852)	0.001
	AT+TT	Ever	9(1.6)	18(3.9)	6.907(2.915-16.366)	0.001
rs11564004	GT+GG	Never	178(32.0)	96(20.6)	1(ref.)	
	TT	Never	226(40.6)	134(28.8)	1.125(0.805-1.573)	0.490
	GT+GG	Ever	67(12.0)	96(20.6)	5.226(3.240-8.428)	0.001
	TT	Ever	86(15.4)	140(30.0)	6.094(3.870-9.596)	0.001

a: adjusted by age, gender.

**Table 7:** Addictive interaction between rs17427875, rs11564004 risk genotypes and smoking exposure.

Measure	rs17427875		rs11564004	
	Estimate	95%CI	Estimate	95%CI
RERI	0.275	-2.846-3.396	0.262	-0.935-1.459
AP	0.075	-0.715-0.864	0.087	-0.298-0.472
S	1.114	0.344-3.609	1.149	0.598-2.210

RERI: relative excess risk due to interaction, AP attributable proportion due to interaction, S synergy index.

**Table 8:** Multiplicative interaction between rs17427875, rs11564004 risk genotypes and smoking exposure.

SNPs	Variables	OR (95%CI)^a^	P
rs17427875	Smoking exposure Rs175427875 interaction	0.860(0.318-2.323)	0.765
rs11564004	Smoking exposure Rs11564004 interaction	1.036(0.608-1.767)	0.895

a: adjusted by age and gender.

## Abbreviation

EMT: epithelial-mesenchymal transition
PDE4D: phosphodiesterase 4D
DOCK8: dedicator of cytokinesis 8
Stat3: signal transducer and activator of transcription 3
EOC: epithelial ovarian cancer
