# Supplementary material for: The Polymorphisms of lncRNA HOXA11-AS and the risk of Lung Cancer in Northeastern Chinese population
Source: J Cancer. 2020 Jan 1;11(3):592–8. doi: 10.7150/jca.35411 (PMC6959036; doi:10.7150/jca.35411)
Supplement: Supplementary file 1 — Supplementary table 1. [file jcav11p0592s1.pdf]

	logFC	logCPM	PValue	FDR
HSPC324	-3.7599	-0.59302	1.57E-157	1.18E-154
FENDRR	-4.00154	3.382938	7.25E-136	3.04E-133
LINC00968	-3.58277	0.578868	2.42E-132	9.14E-130
MIR3945HG	-3.56918	0.028683	2.08E-123	6.91E-121
LINC01082	-4.20824	-2.47087	8.38E-118	2.23E-115
LANCL1-AS1	-3.55297	0.317966	3.17E-104	5.75E-102
HID1-AS1	-2.83264	-1.80538	2.74E-103	4.83E-101
PCAT19	-2.4723	2.855857	9.83E-96	1.43E-93
LINC00656	-3.49248	-2.47738	1.30E-95	1.86E-93
LINC01996	-4.52436	-0.67905	8.89E-91	1.08E-88
LINC02016	-4.93643	-1.0387	1.90E-89	2.23E-87
SMIM25	-2.49666	3.979164	1.89E-87	2.10E-85
MIR22HG	-1.66473	4.573688	9.08E-76	7.65E-74
LINC00163	-3.40541	-2.11548	8.10E-75	6.68E-73
LINC00551	-3.16265	-0.83703	1.51E-68	1.05E-66
LINC01352	-2.38602	-1.80206	5.97E-64	3.68E-62
LINC01936	-2.4453	1.999985	4.01E-63	2.37E-61
LINC00987	-1.74962	0.789179	1.53E-60	8.14E-59
LINC00702	-2.28147	0.856677	2.53E-58	1.25E-56
LINC02014	3.940425	-0.04626	2.31E-57	1.08E-55
LINC01197	-2.05469	-0.32241	3.65E-56	1.62E-54
FAM83A-AS1	6.511362	2.05153	9.20E-56	3.99E-54
LINC02154	-3.36503	0.337945	4.71E-55	2.00E-53
LINC01506	-2.47991	-2.40915	6.74E-55	2.83E-53
TBX2-AS1	-2.07909	0.499454	3.27E-52	1.19E-50
LINC00511	3.134469	4.56428	6.36E-52	2.30E-50
LINC01863	-3.20312	-2.04838	5.27E-51	1.83E-49
LINC01977	4.862941	0.684545	6.78E-51	2.33E-49
LINC01290	-2.19911	-1.56069	2.72E-50	9.08E-49
TBX5-AS1	-1.84987	2.743076	8.71E-50	2.86E-48
PTPRD-AS1	-2.28624	-1.14793	9.31E-50	3.06E-48
AFAP1-AS1	6.307052	6.172697	3.44E-48	1.07E-46
MNX1-AS1	5.2047	0.731498	5.63E-48	1.74E-46
LINC01624	-2.30699	-2.60933	1.34E-45	3.69E-44
MBNL1-AS1	-1.83929	1.988782	2.85E-45	7.72E-44
MED4-AS1	-2.21396	-2.04644	6.62E-45	1.75E-43
PARAL1	-3.02481	0.511802	9.16E-45	2.41E-43
LINC00891	-2.38956	-1.5152	1.98E-44	5.15E-43
PVT1	2.733842	3.902015	2.45E-44	6.32E-43
RUNDC3A-AS1	3.828038	0.782368	1.30E-43	3.26E-42
LINC01270	2.904215	1.744826	8.39E-43	2.04E-41
DDX11-AS1	2.433769	-0.21482	1.10E-42	2.65E-41
PCAT6	2.748534	2.209157	2.80E-42	6.66E-41
MAGI2-AS3	-1.60787	3.954358	3.06E-42	7.28E-41
ZFPM2-AS1	5.284961	1.875456	4.64E-42	1.09E-40
EP300-AS1	-1.76297	-0.4068	2.08E-41	4.73E-40
FOXD3-AS1	7.003029	0.291347	6.83E-41	1.51E-39
DARS-AS1	1.968338	1.33188	9.64E-41	2.12E-39
LINC00857	2.314411	2.061788	1.41E-40	3.09E-39
MIR497HG	-1.43591	-0.04203	1.43E-40	3.12E-39
C10orf25	-1.25174	1.403822	2.60E-40	5.64E-39
FEZF1-AS1	6.223612	2.982081	9.21E-40	1.95E-38
LINC02416	4.169664	-1.11346	1.72E-38	3.48E-37
LINC01572	3.027829	-0.29889	1.87E-38	3.75E-37
LINC02104	-2.18668	-2.67978	5.89E-38	1.16E-36
WWC2-AS2	-1.62955	-0.75526	6.64E-38	1.31E-36
LINC01645	-2.65717	-2.58807	1.26E-37	2.44E-36

MGC27382	-2.97472	-0.3612	1.19E-36	2.21E-35
LINC01703	2.422009	-0.39195	6.00E-36	1.09E-34
LINC00092	-1.89181	-0.29365	2.26E-35	3.97E-34
VPS9D1-AS1	2.856529	2.367709	3.29E-35	5.76E-34
DDN-AS1	2.573528	0.058543	2.31E-34	3.90E-33
WASIR2	3.529567	-0.77095	5.29E-34	8.77E-33
TFAP2A-AS1	3.05721	-0.46406	5.65E-34	9.31E-33
LINC01852	-1.20277	0.420931	8.52E-34	1.40E-32
LINC01271	2.559377	-0.60792	2.29E-32	3.53E-31
LINC02535	4.319841	-0.66167	2.32E-32	3.57E-31
LINC01614	3.753819	0.94335	3.34E-32	5.11E-31
LINC01426	2.568799	2.465949	3.52E-32	5.38E-31
BLACAT1	2.943237	2.331344	1.23E-31	1.82E-30
ELF3-AS1	1.757504	1.360408	1.88E-31	2.76E-30
TDRKH-AS1	2.020431	0.221489	2.00E-31	2.91E-30
ADAMTS9-AS1	-2.27949	0.654071	1.38E-30	1.93E-29
SNHG1	1.589094	5.26328	1.49E-30	2.07E-29
BBOX1-AS1	5.483641	0.92418	1.86E-30	2.57E-29
NAV2-AS2	-2.62202	-2.14162	1.98E-30	2.73E-29
FOXD2-AS1	1.667247	2.142064	2.57E-30	3.53E-29
LINC01963	-1.21294	2.384084	7.84E-30	1.04E-28
LINC02035	-1.0382	3.296928	1.06E-29	1.40E-28
DUBR	-1.25405	1.842821	1.10E-29	1.45E-28
GHET1	1.87854	-0.5104	1.59E-29	2.07E-28
SNHG4	2.248309	1.412679	2.46E-29	3.17E-28
LINC01711	3.785503	-1.60083	9.21E-29	1.16E-27
SBF2-AS1	1.332871	2.423849	9.45E-29	1.19E-27
LINC02332	3.831484	-2.49213	1.87E-28	2.31E-27
SLC2A1-AS1	2.406833	-0.06225	2.00E-28	2.45E-27
CLDN10-AS1	6.387622	0.778388	2.10E-28	2.57E-27
LINC00862	3.824543	-0.39854	3.30E-28	3.99E-27
FGF14-AS2	-1.59833	0.455199	3.76E-28	4.54E-27
CADM3-AS1	-2.16049	-0.45801	4.16E-28	5.00E-27
C1orf220	2.224186	-0.3869	4.33E-28	5.20E-27
LINC00896	3.292284	-0.19393	6.66E-28	7.91E-27
OGFRP1	1.529484	-0.5159	3.83E-27	4.35E-26
LINC02202	-1.36573	-1.40388	4.97E-27	5.63E-26
ATP2B1-AS1	-1.18152	0.649585	5.61E-27	6.32E-26
AGAP11	-2.03883	-2.50831	6.43E-27	7.22E-26
LINC01914	-1.78721	-1.15069	8.10E-27	9.02E-26
NPSR1-AS1	5.80024	-1.40726	1.31E-26	1.44E-25
LINC01460	3.942201	0.389864	1.92E-26	2.10E-25
C2orf48	2.932444	-0.62329	2.61E-26	2.84E-25
LINC02159	4.164647	0.954844	4.80E-26	5.15E-25
SLC12A9-AS1	2.19886	-1.02035	2.29E-25	2.34E-24
DCST1-AS1	1.722633	0.608613	3.41E-25	3.44E-24
SENCR	-1.42739	-0.28153	3.49E-25	3.52E-24
LINC01607	2.536493	-0.12921	4.07E-25	4.09E-24
SH3RF3-AS1	-1.32638	-0.42953	5.00E-25	4.99E-24
LINC00942	6.57361	3.13272	5.28E-25	5.27E-24
EPB41L4A-DT	-1.20658	-0.07891	5.48E-25	5.46E-24
CYP4A22-AS1	2.524087	-1.45831	5.69E-25	5.67E-24
LINC02544	3.528727	-0.71892	5.78E-25	5.75E-24
LINC01705	3.825752	-1.43907	6.25E-25	6.19E-24
KCNMB2-AS1	5.517345	1.086166	6.73E-25	6.65E-24
LINC01833	6.47492	0.684089	2.04E-24	1.95E-23
SNHG3	1.564402	4.958312	3.45E-24	3.25E-23
HOXC-AS2	5.037491	-0.61637	4.18E-24	3.92E-23

DGCR9	3.06821	0.882622	4.44E-24	4.15E-23
LINC00629	3.433663	-2.06453	6.12E-24	5.67E-23
LINC02362	2.60314	1.370867	6.47E-24	5.99E-23
TMPO-AS1	1.583719	1.327416	7.48E-24	6.89E-23
LINC01012	1.990138	-0.26475	1.55E-23	1.40E-22
LINC01836	-1.63581	0.456533	2.10E-23	1.88E-22
C22orf34	-1.24165	1.135587	2.45E-23	2.19E-22
LINC00525	3.44862	-1.2457	4.00E-23	3.53E-22
LINC02580	-1.40923	-2.35219	4.04E-23	3.56E-22
LINC01395	5.053403	-1.34035	4.77E-23	4.18E-22
LINC00628	3.643899	-2.71959	5.46E-23	4.76E-22
LINC00880	3.397632	-0.65788	7.23E-23	6.27E-22
LINC02473	4.090855	-2.47646	7.90E-23	6.84E-22
TMEM51-AS1	1.768146	1.370798	1.33E-22	1.13E-21
LINC02185	-1.92259	-0.52402	1.58E-22	1.34E-21
LINC01765	-2.62828	-1.23921	1.80E-22	1.52E-21
LINC00665	2.34288	4.527694	2.03E-22	1.70E-21
PTPRG-AS1	1.811718	0.717646	2.10E-22	1.76E-21
LINC00887	3.862629	-0.82486	3.01E-22	2.50E-21
RAMP2-AS1	-1.76334	0.578191	3.47E-22	2.87E-21
FRGCA	3.876262	-1.40796	4.12E-22	3.38E-21
LINC01588	1.235674	1.091712	4.66E-22	3.81E-21
LINC02003	4.185194	-2.41094	4.99E-22	4.07E-21
LINC00337	2.92763	-0.4864	5.52E-22	4.49E-21
LINC02474	4.817309	1.179969	6.57E-22	5.32E-21
LINC02081	2.906097	0.412947	7.44E-22	6.01E-21
LINC00461	4.209858	-1.94572	7.44E-22	6.01E-21
STK32A-AS1	3.311153	0.281066	8.43E-22	6.78E-21
MYOSLID	3.37465	0.85533	8.59E-22	6.91E-21
RHPN1-AS1	1.607673	1.338409	9.93E-22	7.94E-21
LINC00160	3.723592	-1.38314	1.27E-21	1.01E-20
FLJ34503	-2.28629	-2.65407	1.36E-21	1.08E-20
LINC01150	-1.45801	-1.9752	3.10E-21	2.42E-20
LINC00115	1.48602	-0.23016	3.54E-21	2.75E-20
ZNF252P-AS1	2.003102	-0.95627	3.90E-21	3.02E-20
LINC00472	-1.85987	0.011713	5.50E-21	4.20E-20
LINC01569	1.50376	1.819723	5.52E-21	4.22E-20
LINC01983	3.780947	-0.28311	1.47E-20	1.08E-19
LINC01348	2.676156	1.16095	2.61E-20	1.88E-19
LINC02265	-1.99423	-1.97065	2.77E-20	1.99E-19
LINC00908	-1.64277	-0.32143	2.80E-20	2.01E-19
USP30-AS1	-1.30261	0.492452	2.99E-20	2.15E-19
PRRT3-AS1	1.791677	0.498295	3.61E-20	2.57E-19
LINC01208	4.179625	-2.69882	5.26E-20	3.70E-19
LINC01827	-2.02657	-2.66387	6.87E-20	4.79E-19
LINC01993	2.887881	-0.81061	7.81E-20	5.42E-19
LINC00624	2.494365	0.689983	8.84E-20	6.10E-19
LINC01415	-1.54969	-1.09343	9.51E-20	6.55E-19
RMDN2-AS1	2.651544	-0.70159	1.06E-19	7.30E-19
PGM5P4-AS1	-1.91308	-2.6231	1.34E-19	9.10E-19
MNX1-AS2	2.981416	-1.28012	1.53E-19	1.04E-18
LINC02560	3.008168	0.634193	1.62E-19	1.10E-18
UCA1	5.605306	2.573123	1.65E-19	1.12E-18
F11-AS1	-2.24391	-1.26012	2.07E-19	1.39E-18
LINC01719	1.561426	0.620529	2.65E-19	1.76E-18
TRIM31-AS1	2.311699	-0.6194	3.16E-19	2.08E-18
CAPN10-DT	1.277558	1.384691	3.27E-19	2.15E-18
CASC16	4.281596	-1.91496	3.91E-19	2.56E-18

LINC01842	3.484839	-0.89546	4.24E-19	2.77E-18
VAC14-AS1	2.601503	-0.71827	7.65E-19	4.90E-18
CALML3-AS1	3.467622	-0.19701	8.79E-19	5.59E-18
BARX1-DT	6.990745	-0.59773	9.36E-19	5.94E-18
SATB2-AS1	3.052916	-2.30432	9.63E-19	6.10E-18
LOXL1-AS1	1.299888	2.345089	1.28E-18	8.05E-18
LINC02576	2.255551	-0.8435	1.68E-18	1.04E-17
PCAT7	2.402268	-0.70556	1.86E-18	1.16E-17
DUXAP8	2.598544	0.95369	2.18E-18	1.35E-17
GAS1RR	-1.48276	-1.2903	2.45E-18	1.51E-17
LINC01140	-1.14048	0.120899	2.47E-18	1.52E-17
MAP3K14-AS1	1.09562	2.230849	2.92E-18	1.79E-17
ITGB1-DT	3.752872	-0.36981	2.98E-18	1.82E-17
RNF144A-AS1	2.154328	0.770701	3.08E-18	1.88E-17
LINC01354	-1.80267	-1.35396	3.16E-18	1.92E-17
LINC01559	6.298213	1.290098	3.62E-18	2.20E-17
LINC00460	4.338017	1.501153	3.64E-18	2.21E-17
MELTF-AS1	1.560469	0.957924	3.88E-18	2.34E-17
LUCAT1	3.196766	2.396497	4.72E-18	2.83E-17
LINC02323	2.939135	-0.5503	4.98E-18	2.98E-17
LINC00884	1.619985	1.235393	5.01E-18	3.00E-17
MAFG-DT	1.248592	2.775207	6.33E-18	3.76E-17
STAU2-AS1	2.122558	-0.60124	6.89E-18	4.08E-17
LINC01537	-1.56053	-1.57784	7.42E-18	4.38E-17
UBE2Q1-AS1	1.866399	-1.83137	7.71E-18	4.55E-17
LINC01268	-1.32129	-0.15325	8.10E-18	4.76E-17
HOTAIR	6.780746	0.065982	8.28E-18	4.86E-17
LINC01303	2.454326	-0.75534	8.90E-18	5.22E-17
LINC00467	1.054761	3.855129	9.94E-18	5.81E-17
LINC01311	1.506487	-0.63857	1.11E-17	6.48E-17
CASC9	5.413611	1.86865	1.13E-17	6.58E-17
LINC01515	1.816355	1.315915	1.35E-17	7.80E-17
UMODL1-AS1	-2.04605	-0.48261	1.80E-17	1.04E-16
LINC02086	3.894377	0.111282	1.82E-17	1.05E-16
LINC00866	2.710117	-2.46496	2.08E-17	1.18E-16
CYTOR	1.25234	3.906243	2.24E-17	1.27E-16
C20orf197	3.065863	1.382427	2.32E-17	1.31E-16
LINC01169	-2.1342	-2.2158	2.45E-17	1.39E-16
STEAP2-AS1	3.254963	-2.69707	2.63E-17	1.48E-16
MYO16-AS1	-2.45921	-0.45161	3.57E-17	1.99E-16
DLEU7-AS1	2.003269	-1.39022	3.57E-17	1.99E-16
HOXC13-AS	5.896239	-1.10278	3.75E-17	2.09E-16
PKP4-AS1	2.500223	0.595959	4.40E-17	2.44E-16
C1orf147	1.839632	-1.34053	4.90E-17	2.70E-16
LINC01232	1.459136	2.353406	4.93E-17	2.72E-16
DRAIC	3.98597	3.341263	5.27E-17	2.90E-16
LINC02163	5.905637	-1.7483	5.87E-17	3.22E-16
LINC00622	-1.26284	-0.85498	7.49E-17	4.09E-16
TRPM2-AS	3.447019	0.319341	9.43E-17	5.11E-16
VWA8-AS1	-1.67427	-2.30678	9.90E-17	5.36E-16
LINC02122	4.655321	-1.53848	9.95E-17	5.38E-16
MIR4435-2HG	1.051872	4.478125	1.29E-16	6.95E-16
SALRNA1	2.925417	-1.175	1.62E-16	8.65E-16
LINC00519	2.416834	-1.55219	1.75E-16	9.28E-16
LINC02133	4.263603	-1.14514	1.76E-16	9.32E-16
LINC01597	3.797171	0.919295	1.78E-16	9.45E-16
GATA6-AS1	-1.7341	0.185564	1.82E-16	9.65E-16
LINC00858	5.01222	0.342841	1.84E-16	9.73E-16

NAALADL2-AS	5.623232	-0.80424	1.87E-16	9.87E-16
PTGES2-AS1	2.390595	-1.97233	1.94E-16	1.02E-15
LINC00501	5.392139	-0.7614	2.00E-16	1.05E-15
MIR4713HG	4.19266	-2.3484	2.11E-16	1.11E-15
LINC02029	4.036022	-2.31806	2.12E-16	1.12E-15
LINC00973	6.080745	0.807875	2.40E-16	1.26E-15
KLHL7-DT	1.765095	0.348437	2.79E-16	1.45E-15
RNF139-AS1	1.39048	1.263847	3.75E-16	1.93E-15
PIK3CD-AS2	1.709419	1.681119	4.08E-16	2.10E-15
LINC02313	4.348442	-1.7187	4.43E-16	2.27E-15
ZKSCAN2-DT	1.478375	1.243611	4.64E-16	2.37E-15
CASC8	3.231889	0.006716	5.04E-16	2.56E-15
FUT8-AS1	1.446875	-0.12727	5.38E-16	2.73E-15
LINC02321	2.579175	-1.7573	6.75E-16	3.39E-15
LINC01214	5.999757	-1.27538	7.29E-16	3.65E-15
MAFA-AS1	4.263729	-1.46639	7.60E-16	3.80E-15
LINC01547	1.563316	2.378998	7.69E-16	3.84E-15
DLX6-AS1	4.667509	0.727672	7.85E-16	3.92E-15
LINC00707	4.497372	0.602957	8.00E-16	3.99E-15
LINC01748	4.812166	-0.13304	8.19E-16	4.08E-15
LINC02033	-1.31101	-2.4807	9.11E-16	4.52E-15
LINC01561	3.126476	-2.18631	9.27E-16	4.60E-15
GAS6-DT	-1.19754	0.139597	1.01E-15	4.99E-15
SFTA1P	-1.77574	4.675636	1.03E-15	5.08E-15
KDM4A-AS1	1.202868	0.183953	1.12E-15	5.50E-15
FIRRE	2.539573	0.045164	1.23E-15	6.06E-15
LINC00471	1.203645	-0.8286	1.36E-15	6.67E-15
LINC01970	2.069794	-1.86855	1.40E-15	6.84E-15
LINC01979	2.351909	-1.59634	1.40E-15	6.86E-15
LINC02588	5.773125	-0.32629	1.60E-15	7.78E-15
FAM222A-AS1	2.664914	-1.26885	1.71E-15	8.28E-15
LINC00165	-2.53083	-2.67521	1.98E-15	9.51E-15
TTLL11-IT1	3.213006	-1.98196	2.06E-15	9.89E-15
SNHG17	1.00151	4.401618	2.55E-15	1.21E-14
LHFPL3-AS2	-2.02568	3.166274	2.91E-15	1.38E-14
LINC02475	4.039776	-0.56843	2.97E-15	1.41E-14
HAGLROS	2.529365	-0.23326	3.08E-15	1.46E-14
LNCOC1	1.762193	-0.60111	3.25E-15	1.53E-14
LINC02285	-1.12873	-1.55537	4.35E-15	2.03E-14
SEMA3B-AS1	-1.21433	-0.94868	4.38E-15	2.04E-14
ATP2A1-AS1	1.621199	-0.74613	4.57E-15	2.13E-14
ELFN1-AS1	3.800862	-0.21547	5.24E-15	2.43E-14
LINC01615	2.230245	-1.23935	5.46E-15	2.52E-14
HOXC-AS3	5.786893	-1.01969	5.54E-15	2.56E-14
LINC00924	-1.30669	-0.90999	5.58E-15	2.57E-14
LINC01436	3.297076	3.132312	5.99E-15	2.76E-14
LYRM4-AS1	1.05744	0.377359	7.82E-15	3.57E-14
ADAMTS9-AS2	-1.45143	0.535099	1.06E-14	4.79E-14
C10orf91	2.190394	-0.52356	1.08E-14	4.86E-14
ERVH48-1	4.66886	2.359191	1.25E-14	5.64E-14
LINC00491	5.610806	-0.78576	1.39E-14	6.22E-14
SNHG25	1.53024	-0.65499	1.44E-14	6.44E-14
HCG15	1.436579	-0.75251	1.46E-14	6.54E-14
LINC02471	-2.19367	0.211083	1.63E-14	7.25E-14
TMEM92-AS1	1.550237	0.060339	1.84E-14	8.19E-14
LINC01355	1.438836	2.548666	1.88E-14	8.35E-14
LNCAROD	5.725174	-1.18033	1.91E-14	8.47E-14
MIR137HG	5.135108	-1.59448	2.04E-14	9.03E-14

LINC01213	4.123002	-2.57255	2.20E-14	9.71E-14
SAMD12-AS1	1.299437	-0.65182	2.57E-14	1.13E-13
POU6F2-AS2	6.231962	-1.36526	2.63E-14	1.16E-13
LINP1	4.165192	0.288157	2.66E-14	1.16E-13
THRA1/BTR	4.872081	-1.53096	2.76E-14	1.21E-13
GSEC	1.310999	2.217648	3.04E-14	1.32E-13
LINC01138	1.214931	2.073408	3.06E-14	1.33E-13
LACTB2-AS1	1.831052	0.027436	3.13E-14	1.36E-13
LINC01513	-1.99432	-2.29545	3.33E-14	1.45E-13
TAF1A-AS1	1.081055	0.67848	3.39E-14	1.47E-13
LINC02158	-1.42753	-2.31002	3.41E-14	1.48E-13
EGOT	2.562339	-0.40465	3.43E-14	1.49E-13
SLC9A3-AS1	1.715904	3.441076	3.45E-14	1.50E-13
SNHG12	1.044558	4.040086	3.46E-14	1.50E-13
LINC00682	5.540686	-2.57609	3.76E-14	1.62E-13
NARF-AS1	1.88596	-2.22807	3.86E-14	1.67E-13
RHOXF1-AS1	-1.66744	1.938075	3.90E-14	1.68E-13
LINC02418	7.603185	1.591073	4.24E-14	1.83E-13
SRGAP3-AS2	-2.36415	1.560341	4.28E-14	1.84E-13
MIR9-3HG	2.716462	2.077124	4.31E-14	1.85E-13
LINC00920	-1.0376	0.498974	4.54E-14	1.95E-13
FLJ33534	1.923711	-2.13013	4.76E-14	2.04E-13
LINC01134	1.489005	-0.05272	5.88E-14	2.50E-13
LINC01234	5.335835	0.347602	6.33E-14	2.69E-13
FLJ12825	2.078429	-1.30179	6.78E-14	2.87E-13
FLJ45513	1.690101	1.083209	6.78E-14	2.87E-13
LINC01611	5.643381	-2.55383	6.97E-14	2.94E-13
LINC02562	2.248724	1.68648	7.32E-14	3.09E-13
LINC01393	2.042969	-1.40103	7.37E-14	3.11E-13
HHATL-AS1	-2.10411	-2.5925	8.02E-14	3.37E-13
LINC01564	2.917395	-0.35065	8.41E-14	3.53E-13
LINC00941	3.220794	0.553062	8.90E-14	3.73E-13
LINC01587	2.990814	-1.70213	9.15E-14	3.83E-13
LINC01287	5.644984	0.603444	1.06E-13	4.42E-13
LINC02345	-1.36101	1.244753	1.09E-13	4.52E-13
SRD5A3-AS1	1.430036	1.663439	1.09E-13	4.53E-13
LINC01305	5.141672	-2.02681	1.24E-13	5.15E-13
LINC01106	1.277617	1.01513	1.32E-13	5.47E-13
LINC02398	3.530847	-1.79876	1.43E-13	5.91E-13
LMO7DN	-1.46908	-2.08265	1.66E-13	6.77E-13
LINC02015	2.619331	0.079516	1.71E-13	6.98E-13
HOXA11-AS	4.336744	-1.28553	2.07E-13	8.41E-13
LINC02387	3.360537	-1.74443	2.28E-13	9.23E-13
NALCN-AS1	-1.78995	-1.83075	2.29E-13	9.27E-13
TMEM147-AS1	1.030838	2.876103	2.62E-13	1.05E-12
LINC01518	5.909481	-2.00124	3.14E-13	1.25E-12
LINC02428	4.308669	-2.38642	3.40E-13	1.35E-12
DPH6-DT	-1.15076	-2.1425	3.42E-13	1.36E-12
LINC01389	1.491813	-1.43219	3.53E-13	1.40E-12
LINC01267	-1.67359	-1.35589	3.65E-13	1.45E-12
MIR503HG	1.735481	0.96329	3.88E-13	1.53E-12
LINC00535	-1.3357	-1.63815	4.01E-13	1.58E-12
UBXN10-AS1	1.789162	0.016475	4.11E-13	1.62E-12
LINC01843	2.198637	0.27281	4.15E-13	1.64E-12
HHIP-AS1	-1.57997	2.101328	4.20E-13	1.65E-12
LINC01484	1.87957	-0.00927	4.66E-13	1.83E-12
LINC02280	1.828862	-2.68433	4.68E-13	1.84E-12
DPP10-AS1	3.385407	0.714901	4.76E-13	1.87E-12

APCDD1L-DT	3.090358	-0.47821	5.11E-13	2.00E-12
LINC01876	2.308081	0.499844	6.25E-13	2.43E-12
NKAIN3-IT1	4.520332	-0.42759	6.97E-13	2.70E-12
STPG3-AS1	2.091908	-0.63072	8.11E-13	3.13E-12
LEMD1-AS1	2.400867	-1.76157	8.60E-13	3.31E-12
LINC00607	-1.36223	0.75439	8.68E-13	3.34E-12
PRMT5-AS1	1.256551	-1.24221	9.12E-13	3.50E-12
LINC01978	1.91329	-2.01643	9.59E-13	3.67E-12
ITGB2-AS1	1.676167	2.476113	1.03E-12	3.93E-12
LINC02365	3.38779	-1.39099	1.05E-12	4.01E-12
ADIRF-AS1	-1.13633	2.693961	1.08E-12	4.11E-12
LINC00944	1.876557	-0.34968	1.14E-12	4.35E-12
LINC02585	1.249562	-2.18349	1.21E-12	4.59E-12
KCTD21-AS1	1.032549	0.934089	1.22E-12	4.63E-12
IGFL2-AS1	4.045778	-0.95264	1.26E-12	4.78E-12
LINC00676	9.946541	2.094369	1.54E-12	5.81E-12
LINC02006	-1.37408	-1.1864	1.56E-12	5.87E-12
LINC00567	3.812855	-1.11657	1.58E-12	5.95E-12
MIR548XHG	6.522065	-1.66883	1.62E-12	6.09E-12
SYNPR-AS1	2.075984	-0.0732	1.71E-12	6.42E-12
ERVE-1	-1.21541	0.450869	1.77E-12	6.64E-12
LINC00582	1.914219	-1.46097	1.78E-12	6.67E-12
LINC00639	-1.28242	-0.32559	1.81E-12	6.76E-12
SRRM2-AS1	1.151775	0.368493	1.82E-12	6.81E-12
LINC01366	-1.27469	-2.72492	1.84E-12	6.87E-12
LINC00365	-1.34332	-0.97701	1.86E-12	6.94E-12
LINC02257	2.271915	1.457765	1.87E-12	6.98E-12
C8orf31	1.529272	1.466082	1.90E-12	7.09E-12
HOXC-AS1	3.208416	-1.81769	1.92E-12	7.17E-12
LINC01281	1.829871	-1.66133	2.00E-12	7.44E-12
LINC02099	1.555686	-1.87094	2.16E-12	8.02E-12
HPN-AS1	1.700537	-0.9378	2.19E-12	8.11E-12
GCC2-AS1	1.002752	-1.734	2.24E-12	8.29E-12
LINC01535	2.039803	0.083002	2.25E-12	8.34E-12
EXTL3-AS1	1.592643	-0.68128	2.25E-12	8.34E-12
BCAR4	6.449913	-0.07833	2.34E-12	8.67E-12
MIR3150BHG	2.175394	-2.04131	2.37E-12	8.78E-12
PANCR	5.673597	-2.5012	2.57E-12	9.48E-12
CHKB-DT	1.069943	-0.3336	2.59E-12	9.52E-12
LINC01804	5.097392	-1.70068	2.69E-12	9.90E-12
PLAC4	4.545434	2.603331	2.88E-12	1.06E-11
LINC00265	1.003782	2.317073	3.62E-12	1.32E-11
LINC01385	5.274663	-2.7684	3.78E-12	1.37E-11
LINC01136	1.810604	-1.54395	3.89E-12	1.41E-11
DLG3-AS1	1.490419	-2.27667	4.08E-12	1.48E-11
LINC01807	5.008496	-1.92335	4.61E-12	1.66E-11
LINC01816	1.448531	-0.2661	5.42E-12	1.94E-11
LINC02289	-1.38245	-0.33539	5.71E-12	2.04E-11
PRC1-AS1	1.551948	-2.05555	6.24E-12	2.22E-11
SLC12A5-AS1	1.730232	-0.91454	6.79E-12	2.41E-11
MIR2052HG	3.811842	-1.65388	6.82E-12	2.42E-11
MIR210HG	1.132764	2.929764	6.85E-12	2.43E-11
SSTR5-AS1	5.195533	-1.05878	6.99E-12	2.48E-11
LINC01277	-1.13419	-1.98892	9.31E-12	3.27E-11
MIR99AHG	-1.15025	1.05126	9.42E-12	3.31E-11
MIR181A1HG	3.589702	-0.57632	9.83E-12	3.45E-11
LHX1-DT	5.704931	-1.74276	1.02E-11	3.58E-11
LINC00342	1.995426	4.439558	1.05E-11	3.68E-11

LINC01511	5.361837	-0.0369	1.06E-11	3.72E-11
LINC02036	1.979965	0.060302	1.11E-11	3.89E-11
FAM225B	1.350388	-0.70092	1.16E-11	4.04E-11
FBXL19-AS1	1.088944	2.027808	1.24E-11	4.30E-11
ST7-OT4	2.924242	-0.75731	1.25E-11	4.34E-11
ITGA6-AS1	1.184627	-1.24166	1.25E-11	4.35E-11
LINC00315	-1.13643	-2.48272	1.28E-11	4.44E-11
TTC3-AS1	2.008348	-2.34758	1.28E-11	4.44E-11
SMC2-AS1	-1.26448	-1.12019	1.38E-11	4.75E-11
NKILA	1.297282	2.188116	1.40E-11	4.84E-11
LINC01010	-1.32723	-0.58514	1.41E-11	4.85E-11
PGM5P3-AS1	-1.54674	-2.37378	1.45E-11	4.99E-11
LINC01980	6.147366	-0.29076	1.52E-11	5.22E-11
GAS5	1.182923	7.802268	1.57E-11	5.40E-11
LINC02126	-1.21492	-2.06739	1.58E-11	5.44E-11
FLJ22447	2.814576	1.847379	1.59E-11	5.46E-11
ADD3-AS1	-1.02744	-1.60664	1.61E-11	5.54E-11
LINC00996	-1.0408	0.41724	1.67E-11	5.73E-11
STAM-AS1	1.301609	-1.06131	1.83E-11	6.25E-11
LINC00638	1.061554	1.429498	1.87E-11	6.39E-11
FAM181A-AS1	-1.96475	-1.55362	2.04E-11	6.93E-11
APOA1-AS	-1.2946	-2.44257	2.09E-11	7.09E-11
LINC01194	6.1039	-0.52104	2.29E-11	7.74E-11
CA3-AS1	-1.16233	-1.32716	2.38E-11	8.05E-11
LINC02178	5.768735	-2.41761	2.41E-11	8.14E-11
TM4SF1-AS1	1.787605	-0.47075	2.50E-11	8.40E-11
FALEC	1.347879	-2.43203	2.51E-11	8.45E-11
LINC01376	1.061264	0.912122	2.55E-11	8.58E-11
MYCNOS	3.681915	-2.13343	2.59E-11	8.69E-11
LINC00954	1.527344	0.117059	2.67E-11	8.96E-11
ALMS1-IT1	1.241759	0.235265	2.97E-11	9.92E-11
LGALS8-AS1	1.374837	-1.40019	3.26E-11	1.08E-10
LINC01301	1.223498	-0.09458	3.37E-11	1.12E-10
MIR31HG	3.329838	-1.15729	3.37E-11	1.12E-10
LINC02555	-1.86717	1.194387	3.42E-11	1.13E-10
HOXA10-AS	4.228832	-2.77976	3.47E-11	1.15E-10
SLC25A25-AS1	1.176697	3.144696	3.50E-11	1.16E-10
POT1-AS1	1.370322	0.437655	3.52E-11	1.16E-10
ZNF571-AS1	1.763436	0.513072	3.52E-11	1.17E-10
GTF3C2-AS1	1.334571	-1.0053	3.61E-11	1.19E-10
LINC01299	5.531782	-2.17619	3.68E-11	1.21E-10
CCDC13-AS1	-1.04467	-1.78042	3.75E-11	1.24E-10
DPYD-AS1	3.497667	0.635775	3.80E-11	1.25E-10
LINC02492	5.391198	-2.58389	3.84E-11	1.27E-10
LINC00824	4.159331	-0.53745	4.00E-11	1.32E-10
LINC00513	2.275388	0.615293	4.03E-11	1.33E-10
KCNQ1OT1	2.066272	4.132794	4.06E-11	1.34E-10
FLJ16779	2.69648	-0.7011	4.21E-11	1.39E-10
NFYC-AS1	1.06601	0.446789	4.56E-11	1.49E-10
VIPR1-AS1	-1.32279	0.380802	4.93E-11	1.61E-10
HIF1A-AS2	2.418212	-0.13567	5.16E-11	1.68E-10
LINC02109	4.409829	-2.40305	5.30E-11	1.73E-10
DGCR10	2.047238	-2.11517	5.73E-11	1.86E-10
BMS1P4	1.016456	-0.76274	5.92E-11	1.92E-10
ZNF793-AS1	1.423235	0.607436	5.96E-11	1.94E-10
DSCR8	6.404565	-0.64013	6.34E-11	2.05E-10
LINC00853	1.240148	-0.33996	6.44E-11	2.09E-10
TBL1XR1-AS1	3.877241	-2.08602	6.46E-11	2.09E-10

LINC02525	5.672153	-2.13084	6.53E-11	2.11E-10
LINC01269	2.098883	-0.92974	6.57E-11	2.13E-10
RMST	-1.86707	-0.91515	6.59E-11	2.13E-10
LINC02293	4.522586	-2.18613	6.69E-11	2.16E-10
DSCAM-AS1	7.769999	3.913997	6.69E-11	2.16E-10
LEF1-AS1	1.205715	-1.59809	7.35E-11	2.36E-10
MIS18A-AS1	1.35405	-1.95509	7.43E-11	2.39E-10
LINC01344	2.633154	-2.63258	7.61E-11	2.44E-10
ZBTB20-AS1	2.932084	-1.36939	8.68E-11	2.77E-10
LINC02377	5.707993	-2.64851	9.72E-11	3.09E-10
LINC01571	-2.12684	-2.1623	1.03E-10	3.26E-10
LINC01629	3.347474	-1.95138	1.07E-10	3.39E-10
LINC02253	4.940053	-0.72831	1.07E-10	3.40E-10
LINC01600	1.945461	-2.86403	1.09E-10	3.44E-10
LINC00898	4.559136	-2.73071	1.15E-10	3.62E-10
LINC01224	2.601553	0.850021	1.15E-10	3.63E-10
MIR193BHG	1.501286	-0.63002	1.17E-10	3.70E-10
LINC02048	3.905592	-2.79773	1.25E-10	3.94E-10
HOXB-AS4	4.030102	-2.30581	1.37E-10	4.30E-10
MIAT	1.794185	4.44373	1.39E-10	4.35E-10
C15orf54	2.553675	-1.22139	1.46E-10	4.56E-10
PURPL	3.834388	-0.80181	1.52E-10	4.74E-10
LINC00184	2.116381	-1.76228	1.60E-10	4.99E-10
SAMSN1-AS1	3.147029	-2.47317	1.62E-10	5.05E-10
LINC01814	1.336244	0.412385	1.68E-10	5.20E-10
DLEU2	1.027461	2.424324	1.88E-10	5.82E-10
CASC19	4.378587	-1.96477	2.39E-10	7.32E-10
LINC00659	3.606209	-2.47669	2.42E-10	7.41E-10
STRA6LP	-1.01944	-1.7144	2.72E-10	8.30E-10
DSCR9	1.247835	-2.39168	2.95E-10	8.99E-10
C6orf99	1.112314	-0.04869	2.96E-10	9.02E-10
RNF157-AS1	1.217369	1.292623	3.79E-10	1.14E-09
C5orf66-AS1	4.149303	-2.5161	3.89E-10	1.17E-09
LINC00346	1.292968	2.729311	3.99E-10	1.20E-09
LINC00524	3.407471	-2.07822	4.01E-10	1.20E-09
PDXDC2P-NPII	1.067539	2.969462	4.18E-10	1.25E-09
LNCSRLR	2.189467	-1.85695	4.28E-10	1.28E-09
LINC01206	3.748921	-2.03031	4.35E-10	1.30E-09
LINC02012	1.484161	-1.19174	4.73E-10	1.41E-09
LINC02331	2.656905	-2.24241	4.81E-10	1.43E-09
LINC01510	3.643495	-2.53089	4.81E-10	1.44E-09
LINC01485	2.093804	-2.17569	5.03E-10	1.50E-09
LINC00173	1.457617	-0.46845	5.05E-10	1.50E-09
ERVMER61-1	5.571015	-2.50657	5.27E-10	1.56E-09
LINC02195	1.838391	-1.30691	5.61E-10	1.66E-09
RDH10-AS1	1.869689	0.703729	5.63E-10	1.67E-09
KCNQ5-IT1	4.145822	-2.47518	6.34E-10	1.87E-09
LINC02484	5.820775	-2.54393	6.56E-10	1.93E-09
H19	3.095511	6.556737	6.96E-10	2.04E-09
PRKCA-AS1	4.822159	-1.30277	7.14E-10	2.09E-09
PACERR	1.701189	-1.12887	7.31E-10	2.14E-09
LINC01655	2.110793	-0.27406	7.50E-10	2.19E-09
P3H2-AS1	-1.61215	-2.12442	7.50E-10	2.19E-09
UTAT33	1.158368	-1.70066	8.12E-10	2.37E-09
KIF25-AS1	3.271561	0.554868	8.39E-10	2.44E-09
LINC01116	2.018194	1.233531	8.93E-10	2.60E-09
SLX1A-SULT1A	1.413173	-2.58793	9.31E-10	2.70E-09
LINC02407	1.452318	-1.28475	9.37E-10	2.72E-09

LINC00221	5.229548	-0.73531	9.71E-10	2.81E-09
MALAT1	2.394829	9.985126	9.82E-10	2.84E-09
JMJD1C-AS1	1.357291	0.617212	1.33E-09	3.80E-09
LINC00648	3.988453	0.422868	1.36E-09	3.88E-09
LINC01786	1.339344	-1.16841	1.36E-09	3.90E-09
G2E3-AS1	5.146497	-2.24596	1.41E-09	4.02E-09
LINC01238	1.327549	-0.54764	1.41E-09	4.02E-09
LINC00355	4.337461	-0.87404	1.42E-09	4.03E-09
DSCR4	5.893876	-2.51559	1.53E-09	4.35E-09
LINC00592	1.964331	-1.79415	1.56E-09	4.42E-09
LINC00589	2.103659	-1.16763	1.66E-09	4.69E-09
DKFZp779M06!	-1.12345	-1.89684	1.70E-09	4.79E-09
ABCA9-AS1	3.344396	-1.81191	1.76E-09	4.96E-09
SLC8A1-AS1	2.565048	-0.51057	2.09E-09	5.84E-09
LINC01356	2.270311	-1.77692	2.15E-09	6.02E-09
LINC02207	-1.19922	-2.13709	2.21E-09	6.18E-09
C11orf72	1.523662	-2.54887	2.30E-09	6.41E-09
SOX21-AS1	2.197235	1.178524	2.38E-09	6.62E-09
LINC00958	2.455356	1.042456	2.45E-09	6.81E-09
VCAN-AS1	3.476106	-1.75787	2.48E-09	6.90E-09
FAM30A	1.546627	2.562323	2.51E-09	6.99E-09
RBPMS-AS1	-1.03779	1.699319	2.61E-09	7.25E-09
LINC02320	1.685439	-2.17944	2.74E-09	7.60E-09
MIR4527HG	3.492398	-2.41754	2.77E-09	7.69E-09
FLJ31356	1.279167	0.292086	2.85E-09	7.88E-09
LINC00578	1.275627	1.30235	3.02E-09	8.35E-09
MAGEA4-AS1	6.457519	-1.83055	3.04E-09	8.38E-09
EIPR1-IT1	1.597016	-2.09554	3.08E-09	8.50E-09
HNF1A-AS1	2.62581	1.11776	3.10E-09	8.54E-09
CRTC3-AS1	1.033635	-1.75419	3.14E-09	8.66E-09
LINC01913	2.876421	-1.29742	3.15E-09	8.69E-09
ATP11AUN	3.871373	-0.53412	3.25E-09	8.94E-09
MANCR	2.915329	-1.06835	3.30E-09	9.07E-09
LINC02404	5.669711	-1.47211	3.32E-09	9.12E-09
LINC02335	5.114974	-2.88933	3.52E-09	9.62E-09
C12orf80	1.939215	-2.6231	3.71E-09	1.01E-08
LINC01419	6.960806	1.016292	3.79E-09	1.03E-08
LINC00943	1.890444	-1.38732	3.95E-09	1.08E-08
LINC02454	2.8362	-2.05886	5.32E-09	1.43E-08
FOXP1-IT1	3.541289	-0.77783	5.34E-09	1.44E-08
ZFHX4-AS1	3.85185	-2.42272	5.50E-09	1.48E-08
LINC02476	5.466615	-2.01223	6.76E-09	1.81E-08
LINC01123	1.082384	1.068428	7.19E-09	1.92E-08
HAR1B	2.271747	-1.28517	7.53E-09	2.00E-08
MEG3	1.910121	5.134065	7.72E-09	2.05E-08
TARID	-1.34349	0.604018	7.92E-09	2.10E-08
LCMT1-AS2	2.311212	-0.61343	7.97E-09	2.12E-08
TMEM132D-AS	5.534729	-2.12142	8.57E-09	2.27E-08
LINC01132	1.060108	0.708412	8.69E-09	2.30E-08
AP4B1-AS1	1.073246	-0.43817	8.75E-09	2.31E-08
LINC01924	4.683786	-2.81063	8.78E-09	2.32E-08
LINC00330	2.192894	-2.82461	8.79E-09	2.32E-08
SCARNA9	2.432753	0.666362	9.04E-09	2.39E-08
STEAP3-AS1	1.240737	-1.05223	9.08E-09	2.40E-08
LINC00470	3.329535	-0.50491	9.10E-09	2.40E-08
LINC01770	1.033699	1.679357	9.31E-09	2.46E-08
LINC02267	5.364054	-2.35056	9.87E-09	2.60E-08
LINC01605	1.941214	-0.81394	9.92E-09	2.61E-08

DSG1-AS1	4.567502	-2.72309	9.98E-09	2.62E-08
LINC00649	1.069883	3.306649	1.00E-08	2.63E-08
LINC02468	2.615759	-2.61393	1.04E-08	2.73E-08
LINC01667	5.54233	-0.53275	1.05E-08	2.76E-08
CASC15	1.366066	3.107126	1.06E-08	2.76E-08
LINC01249	4.763415	-2.61644	1.09E-08	2.84E-08
TUSC8	3.894721	-0.61726	1.11E-08	2.89E-08
LINC00482	1.257796	1.878405	1.11E-08	2.90E-08
C8orf34-AS1	-1.36638	2.858165	1.12E-08	2.91E-08
LMF1-AS1	-1.14912	-2.06853	1.16E-08	3.02E-08
KIZ-AS1	1.785603	-1.69542	1.26E-08	3.28E-08
DANT2	2.370508	-0.73865	1.28E-08	3.33E-08
LINC01257	4.615867	-1.33265	1.31E-08	3.39E-08
DNAH17-AS1	1.574042	-0.30737	1.34E-08	3.47E-08
LINC00668	3.790234	-0.26633	1.39E-08	3.59E-08
SATB1-AS1	1.221614	-0.11769	1.45E-08	3.74E-08
GDNF-AS1	2.035074	-2.39185	1.63E-08	4.17E-08
FAM230C	5.20988	-2.01058	1.78E-08	4.56E-08
LINC02506	4.700467	-1.4377	1.92E-08	4.90E-08
LINC01126	1.149994	-1.26516	1.99E-08	5.07E-08
MIR3142HG	1.317907	0.91201	2.05E-08	5.20E-08
LINC00393	4.746496	-2.47807	2.05E-08	5.20E-08
LINC01345	5.261534	-2.49984	2.12E-08	5.38E-08
LINC02582	5.688275	-1.68135	2.28E-08	5.77E-08
MIR194-2HG	2.353761	-1.98488	2.30E-08	5.80E-08
LINC01446	4.327074	-0.58107	2.37E-08	5.99E-08
FRMD6-AS1	1.184762	-1.15687	2.41E-08	6.09E-08
LINC01237	1.089455	-1.18943	2.73E-08	6.87E-08
LINC00392	5.586139	-2.76008	2.78E-08	6.98E-08
LINC01185	1.595468	-2.77124	2.91E-08	7.29E-08
LINC02246	1.628022	-1.53247	2.94E-08	7.37E-08
RMRP	6.219473	-0.77421	2.98E-08	7.47E-08
LHFPL3-AS1	-1.66733	-2.20692	3.17E-08	7.92E-08
AQP4-AS1	-1.31436	-0.79804	3.34E-08	8.33E-08
PROX1-AS1	2.263209	-0.34439	3.45E-08	8.60E-08
ACOXL-AS1	-1.07247	-0.37284	3.65E-08	9.06E-08
CAMTA1-DT	1.059405	-2.04682	3.77E-08	9.36E-08
LINC01659	2.078646	-1.08979	4.43E-08	1.09E-07
LINC01121	1.490918	-1.46914	4.73E-08	1.16E-07
LINC02068	1.568394	-1.83802	4.86E-08	1.19E-07
MIR646HG	1.391023	0.922146	5.18E-08	1.27E-07
ARHGAP26-AS	2.619741	-2.41324	5.19E-08	1.27E-07
MACC1-AS1	3.358042	-1.80398	5.55E-08	1.35E-07
WASHC5-AS1	1.31111	-2.09814	6.14E-08	1.49E-07
AGAP1-IT1	-1.0825	-0.95577	7.25E-08	1.75E-07
LINC00543	1.928765	-0.43678	7.41E-08	1.79E-07
LINC01915	-1.00221	-1.71888	7.98E-08	1.92E-07
LINC02041	1.795501	-0.21114	8.16E-08	1.96E-07
LBX1-AS1	-1.11806	-2.60968	8.39E-08	2.02E-07
DENND5B-AS1	1.590493	-2.71434	9.18E-08	2.20E-07
TEX41	1.51574	0.369067	9.59E-08	2.29E-07
SIRPG-AS1	1.913609	-2.26881	1.02E-07	2.43E-07
CASC20	3.563798	-2.21809	1.10E-07	2.61E-07
ARLNC1	1.982752	-1.45321	1.17E-07	2.77E-07
SH3PXD2A-AS	1.692534	0.026368	1.27E-07	3.00E-07
LINC02188	2.688581	0.04806	1.33E-07	3.14E-07
SIRLNT	5.195028	-2.27514	1.35E-07	3.19E-07
LINC01341	1.188003	0.14731	1.38E-07	3.25E-07

GACAT3	4.744217	-2.64002	1.46E-07	3.43E-07
LINC02037	3.695652	-1.92038	1.50E-07	3.51E-07
LINC02327	4.234214	-2.61024	1.57E-07	3.68E-07
CASC11	1.400101	-2.74237	1.70E-07	3.97E-07
IFNG-AS1	1.656317	-0.76758	1.71E-07	3.98E-07
LINC01929	1.8001	-0.01944	1.71E-07	4.00E-07
WDR11-AS1	-1.14221	-1.75266	1.73E-07	4.02E-07
PRR7-AS1	1.118912	-1.00228	1.87E-07	4.33E-07
LINC00632	1.472607	-1.30587	1.92E-07	4.46E-07
LINC01556	1.556032	-1.79422	2.16E-07	4.98E-07
FLJ42969	1.931865	-2.04516	2.24E-07	5.17E-07
LINC02600	-1.22146	-0.72744	2.24E-07	5.17E-07
ST8SIA6-AS1	3.231864	0.535234	2.42E-07	5.57E-07
SLC26A4-AS1	2.206618	0.537962	2.47E-07	5.69E-07
MIR663AHG	2.02979	1.739116	2.56E-07	5.87E-07
LINC00871	3.569576	-2.52198	2.61E-07	5.99E-07
LINC00284	-1.45503	-1.59724	2.63E-07	6.04E-07
LINC01608	5.090141	-2.56759	2.65E-07	6.08E-07
PARD3-AS1	1.235777	-2.23966	2.81E-07	6.44E-07
ST7-AS2	2.800834	-0.73598	3.13E-07	7.14E-07
BASP1-AS1	1.862383	-2.29303	3.33E-07	7.56E-07
LINC01346	4.60081	-2.40445	3.43E-07	7.76E-07
LINC02100	1.332212	-2.14159	3.56E-07	8.05E-07
LINC01079	3.378434	-2.57646	3.60E-07	8.12E-07
DPYD-AS2	4.075627	-1.88955	3.65E-07	8.23E-07
THCAT158	1.164937	-1.11701	3.72E-07	8.38E-07
LINC01740	3.328866	-1.66614	3.88E-07	8.74E-07
LINC01204	2.366166	-2.78631	4.54E-07	1.02E-06
SMCR5	2.304839	-2.15746	4.63E-07	1.04E-06
ASMTL-AS1	1.178705	1.933031	4.64E-07	1.04E-06
FOXP4-AS1	1.095075	-0.43013	5.09E-07	1.14E-06
LINC00637	1.372093	-2.66783	5.13E-07	1.14E-06
NCBP2-AS1	1.00046	-1.74927	6.48E-07	1.43E-06
RBMS3-AS3	-1.00174	-1.55467	6.52E-07	1.44E-06
LINC01992	4.179508	-2.54709	7.24E-07	1.59E-06
LINC00621	1.376738	-2.67367	8.20E-07	1.79E-06
LINC01896	4.630731	-2.76485	8.42E-07	1.84E-06
ZBTB20-AS5	3.783488	-1.56213	9.09E-07	1.98E-06
LINC02542	1.332829	0.589287	9.17E-07	2.00E-06
GPR1-AS	4.389182	-1.9814	1.01E-06	2.19E-06
LINC01594	2.013671	-2.73698	1.06E-06	2.30E-06
LINC01117	1.711172	-2.01674	1.08E-06	2.33E-06
LINC00216	1.432492	-0.87975	1.15E-06	2.49E-06
LINC01444	3.614914	-2.69994	1.19E-06	2.57E-06
RPS6KA2-IT1	1.069663	-0.91291	1.25E-06	2.70E-06
RBAKDN	2.049774	-2.53217	1.35E-06	2.90E-06
TH2LCRR	1.251877	-1.55677	1.43E-06	3.07E-06
LINC01502	2.710944	-1.7436	1.46E-06	3.12E-06
LINC02577	1.998945	1.90438	1.60E-06	3.41E-06
LINC00922	1.923738	0.976415	1.74E-06	3.69E-06
OSBPL10-AS1	2.532062	-2.42351	1.90E-06	4.02E-06
LINC01948	1.01098	-1.75389	1.98E-06	4.19E-06
SND1-IT1	2.339255	-0.9985	2.19E-06	4.60E-06
LINC01695	2.05851	-2.61154	2.20E-06	4.63E-06
LINC01013	-1.05249	-2.46263	2.38E-06	4.99E-06
LINC02381	1.113404	2.577164	2.46E-06	5.14E-06
NFIA-AS1	2.61991	-2.19763	2.50E-06	5.22E-06
THORLNC	1.001067	-1.38934	2.52E-06	5.26E-06

PAQR9-AS1	3.596165	-2.84588	2.74E-06	5.70E-06
LINC01694	1.27863	1.107961	2.86E-06	5.93E-06
LINC02300	2.631241	-0.9605	2.95E-06	6.12E-06
SLC7A11-AS1	1.760022	-0.62915	3.21E-06	6.64E-06
PKIA-AS1	1.90228	-2.26606	3.27E-06	6.77E-06
MIR3681HG	1.879832	-2.03971	3.37E-06	6.96E-06
LINC02595	1.566956	-2.35044	3.40E-06	7.02E-06
LINC02422	1.351224	-1.97267	3.47E-06	7.15E-06
LINC01124	1.034066	1.200245	3.71E-06	7.62E-06
LINC02303	3.932013	-2.46723	3.82E-06	7.84E-06
JARID2-AS1	1.223161	-2.55046	4.12E-06	8.43E-06
DOCK4-AS1	2.800704	-1.52286	4.18E-06	8.54E-06
LINC02266	2.847743	-2.13696	4.52E-06	9.21E-06
LINC02532	1.892975	0.322664	4.69E-06	9.55E-06
MIR133A1HG	2.488349	-1.03888	4.78E-06	9.72E-06
UBE2R2-AS1	1.337338	-2.47639	4.79E-06	9.74E-06
EWSAT1	1.370774	-1.68491	4.81E-06	9.78E-06
LINC01105	-1.45718	-0.57519	5.35E-06	1.08E-05
HOXD-AS2	1.87633	-1.37209	5.84E-06	1.18E-05
LINC01634	1.905393	-2.72341	5.86E-06	1.18E-05
LINC01697	2.422468	-1.87497	5.99E-06	1.21E-05
LINC00540	1.666734	-0.13404	6.13E-06	1.23E-05
LINC00574	1.049318	-2.15527	6.34E-06	1.27E-05
LY6E-DT	1.00735	0.849753	6.35E-06	1.28E-05
ERI3-IT1	1.662897	-2.47327	6.36E-06	1.28E-05
LINC01886	3.935973	-2.37129	6.46E-06	1.30E-05
CYMP-AS1	3.034944	-2.12136	6.63E-06	1.33E-05
ATP11A-AS1	2.572145	-0.88469	6.87E-06	1.38E-05
IGF2-AS	2.461375	-2.10844	7.39E-06	1.47E-05
SCAANT1	1.022741	-2.6009	8.07E-06	1.60E-05
C1orf195	2.066896	-2.41201	8.14E-06	1.62E-05
MIR7-3HG	3.251209	-2.28017	8.40E-06	1.67E-05
LINC01579	1.536874	0.301633	8.42E-06	1.67E-05
LINC02385	3.605987	-2.56144	8.74E-06	1.73E-05
LINC00336	1.259034	-2.02115	8.77E-06	1.73E-05
FSIP2-AS1	1.248884	-2.71034	9.04E-06	1.79E-05
MAGI1-IT1	1.672179	-1.1429	9.06E-06	1.79E-05
PCAT18	2.36067	-1.7075	9.81E-06	1.93E-05
LINC02487	1.568427	-2.08658	9.99E-06	1.96E-05
LINC02470	3.069051	-2.35494	1.05E-05	2.07E-05
SLCO4A1-AS1	1.625433	0.394557	1.24E-05	2.42E-05
LINC01658	1.901352	-2.60415	1.24E-05	2.42E-05
LINC01322	2.846021	-2.13852	1.25E-05	2.43E-05
CHL1-AS2	1.569022	-1.51748	1.32E-05	2.57E-05
MPRIP-AS1	1.869761	-2.45956	1.34E-05	2.61E-05
TTLL10-AS1	-1.12124	-1.32813	1.39E-05	2.70E-05
TCL6	1.695617	-0.97539	1.52E-05	2.93E-05
ANKRD44-IT1	2.066629	-1.08698	1.55E-05	3.00E-05
CACNA1G-AS1	1.18061	-2.76853	1.63E-05	3.14E-05
LINC02315	2.120224	-0.68119	1.86E-05	3.56E-05
LINC01730	1.118781	-0.81121	1.86E-05	3.57E-05
MAL2-AS1	1.290643	-0.60085	1.87E-05	3.58E-05
BEAN1-AS1	1.072855	-2.36307	1.97E-05	3.76E-05
MIR205HG	1.864226	2.435519	2.05E-05	3.91E-05
ARHGEF38-IT1	1.313805	-1.52259	2.09E-05	3.98E-05
ROR1-AS1	1.15659	-0.89706	2.10E-05	4.00E-05
LINC01433	2.110408	-2.53103	2.20E-05	4.18E-05
UPK1A-AS1	1.905576	-2.80364	2.48E-05	4.69E-05

RFPL1S	1.207666	-0.40925	2.63E-05	4.96E-05
LINC02458	1.537208	-1.38017	2.98E-05	5.59E-05
LINC00514	1.042704	-0.71026	2.99E-05	5.61E-05
LINC01907	1.039214	-2.73589	3.01E-05	5.64E-05
LINC00930	-1.13515	-0.3709	3.02E-05	5.65E-05
LINC01554	1.195743	-1.00938	3.11E-05	5.83E-05
CASC18	1.30285	-1.76395	3.15E-05	5.89E-05
KCCAT333	1.47665	0.608739	3.19E-05	5.96E-05
LINC01612	-1.25666	-0.70436	3.28E-05	6.13E-05
LINC01342	1.379795	-2.1082	3.35E-05	6.25E-05
PART1	2.08234	0.468831	3.38E-05	6.30E-05
LINC01055	1.126629	-1.89728	3.46E-05	6.44E-05
LINC01781	1.181521	-1.00935	3.46E-05	6.45E-05
OVCH1-AS1	-1.06429	-2.7017	3.68E-05	6.83E-05
DLGAP1-AS5	2.427357	-1.09947	3.80E-05	7.05E-05
CYP4F26P	1.58307	-1.70816	4.29E-05	7.91E-05
PCCA-AS1	1.833127	-2.33501	4.92E-05	9.02E-05
LINC02446	1.16706	0.577562	5.14E-05	9.41E-05
LINC01163	1.959092	-2.50099	5.69E-05	0.000104
IGF2BP2-AS1	2.025585	-2.13944	5.72E-05	0.000104
MEG9	1.503942	-1.35076	5.75E-05	0.000105
LINC01060	1.949303	-2.30763	5.79E-05	0.000105
LINC02334	1.247352	-1.4949	5.83E-05	0.000106
LINC00323	1.095848	-1.57448	6.23E-05	0.000113
LINC02551	1.30329	-2.24098	6.90E-05	0.000125
KCNMA1-AS1	1.483717	-2.41639	7.46E-05	0.000134
LINC02397	1.15078	-0.94224	7.80E-05	0.00014
LINC00840	-1.05788	-2.2557	8.14E-05	0.000146
BTBD9-AS1	2.092155	-1.76515	8.99E-05	0.00016
PSORS1C3	1.529916	0.059567	9.64E-05	0.000171
LINC01443	2.375156	-2.49649	9.90E-05	0.000176
TLR8-AS1	1.697298	-0.52682	9.91E-05	0.000176
LINC00664	1.24533	0.271243	0.0001	0.000178
NADK2-AS1	1.031904	-1.58671	0.000105	0.000185
LINC01423	1.498772	-2.37431	0.00011	0.000194
LINC00494	1.165264	-0.93855	0.000113	0.000199
LINC01018	1.182644	1.617268	0.000124	0.000218
LINC01785	-1.81277	-2.27762	0.000136	0.000238
DIAPH2-AS1	1.087309	-1.21675	0.000154	0.000269
MRTFA-AS1	1.459007	-1.13471	0.00016	0.000279
LINC00200	3.109939	-1.93659	0.000168	0.000293
MEG8	1.615973	-2.22907	0.000182	0.000314
SLC5A4-AS1	1.058948	-2.4544	0.000198	0.000342
ATP13A4-AS1	-1.19436	0.041019	0.000215	0.000369
LSINCT5	-1.14111	-2.37561	0.000224	0.000384
TRHDE-AS1	-1.18154	-0.65929	0.000228	0.000391
LINC01940	1.884414	-2.78535	0.000238	0.000407
LINC01133	1.601726	1.444258	0.000243	0.000416
PRKG1-AS1	1.236623	-1.93842	0.000244	0.000416
MIR4500HG	1.519525	-2.03207	0.000267	0.000454
LINC00479	1.244154	-2.58794	0.00027	0.000459
KC6	1.699166	-0.93575	0.000271	0.00046
LINC02055	1.919326	-1.85537	0.000288	0.000487
RSF1-IT2	1.010133	-2.40593	0.000298	0.000504
C9orf41-AS1	1.05954	-2.63755	0.000308	0.00052
C5orf17	1.898933	-0.32534	0.000311	0.000525
PICSAR	-1.24288	-0.85477	0.000313	0.000528
ZNF350-AS1	1.065066	-0.56915	0.000407	0.000679

LUNAR1	1.002676	-2.59455	0.000438	0.000728
LINC01139	1.399258	0.598005	0.000457	0.000759
EMX2OS	1.926791	-1.11571	0.00046	0.000762
HORMAD2-AS	1.232391	0.25466	0.000484	0.000801
PRICKLE2-AS3	1.772333	-2.26274	0.000495	0.000817
ITPK1-AS1	1.796117	-2.37085	0.000512	0.000844
CDC42-IT1	1.235769	-2.34667	0.000534	0.000878
LINC02351	1.572594	-2.26445	0.000534	0.000878
EPN2-AS1	1.233286	-1.95131	0.000582	0.000952
LINC02241	2.501353	-0.98535	0.000647	0.001052
LINC00939	1.666068	-1.96082	0.000692	0.001122
PHEX-AS1	1.770677	-2.64862	0.000703	0.001138
PTCSC3	1.02196	0.614733	0.000948	0.001513
LINC01235	1.018061	-0.26968	0.000972	0.00155
LINC01819	1.56829	-1.28462	0.00106	0.001683
B3GALT5-AS1	1.577668	-1.34883	0.001064	0.00169
LINC00605	1.095197	-1.86474	0.001198	0.001892
DLGAP1-AS3	1.717852	-2.59969	0.001609	0.002504
RBMS3-AS2	1.476361	-1.35004	0.00165	0.002564
LINC01108	-1.0619	0.313957	0.002176	0.003334
LINC01606	1.682924	-2.00783	0.002524	0.003842
NBAT1	1.070128	-1.6778	0.003528	0.005271
MRPL23-AS1	1.200399	-2.34434	0.004818	0.00709
TMEM108-AS1	1.840658	-1.74546	0.005229	0.007663
FAM41C	1.366141	-0.69462	0.011447	0.016039
FAM53B-AS1	1.001527	-2.47965	0.01892	0.025828
