# Supplementary material for: The Polymorphisms of lncRNA HOXA11-AS and the risk of Lung Cancer in Northeastern Chinese population
Source: J Cancer. 2020 Jan 1;11(3):592–8. doi: 10.7150/jca.35411 (PMC6959036; doi:10.7150/jca.35411)
Supplement: Supplementary file 2 — Supplementary table 2. [file jcav11p0592s2.pdf]

	logFC	logCPM	PValue	FDR
LANCL1-AS1	-4.13253	-0.32062	9.42E-269	2.56E-265
LINC00968	-4.38231	0.266771	5.56E-185	1.51E-182
HSPC324	-4.38258	-1.23003	3.32E-178	7.72E-176
ADAMTS9-AS2	-3.5672	-0.7012	1.66E-171	3.51E-169
HID1-AS1	-4.0229	-2.27724	4.48E-166	8.70E-164
MIR3945HG	-4.26085	-0.41427	7.79E-163	1.40E-160
LINC01082	-4.48369	-2.97656	2.05E-140	2.27E-138
SFTA1P	-4.61897	3.132543	5.09E-139	5.44E-137
LINC02016	-6.2851	-1.59465	3.56E-137	3.70E-135
FENDRR	-4.05736	3.106083	3.27E-134	3.23E-132
SMIM25	-3.05525	3.501118	2.90E-130	2.52E-128
ADAMTS9-AS1	-3.94159	-0.13756	8.83E-130	7.60E-128
PCAT19	-2.97239	2.46493	1.04E-120	7.19E-119
LINC00891	-3.46347	-2.16893	2.65E-111	1.49E-109
KCNMB2-AS1	7.430097	3.226876	4.97E-110	2.73E-108
LINC01936	-3.4457	1.3455	5.36E-106	2.74E-104
LINC00607	-3.03486	-0.50016	3.16E-102	1.47E-100
GATA6-AS1	-3.2108	-0.91623	4.32E-100	1.93E-98
LINC00702	-3.10119	0.535825	1.72E-99	7.58E-98
LHFPL3-AS2	-4.23113	1.820653	3.49E-99	1.52E-97
BBOX1-AS1	6.837164	2.685711	1.74E-95	6.96E-94
RBPMS-AS1	-2.85549	0.3885	3.97E-95	1.58E-93
MIR497HG	-2.07665	-0.34965	1.02E-92	3.96E-91
TBX5-AS1	-2.676	2.269161	1.69E-86	5.77E-85
CASC9	7.45081	3.675762	2.17E-86	7.35E-85
LINC00511	4.043807	5.38328	3.78E-85	1.21E-83
LINC01852	-1.80998	-0.16187	4.41E-83	1.35E-81
ACOXL-AS1	-2.64997	-1.76456	1.96E-81	5.76E-80
LINC01290	-2.38755	-1.92243	8.65E-81	2.52E-79
LINC01352	-2.8467	-2.19328	5.18E-80	1.49E-78
MIR22HG	-1.96065	4.458873	1.94E-79	5.52E-78
LINC01197	-2.62892	-0.60692	1.16E-78	3.23E-77
LINC01572	3.318882	0.278065	4.05E-77	1.08E-75
LINC01996	-4.82944	-1.21949	1.53E-75	3.95E-74
LINC02555	-3.98518	0.093427	2.14E-75	5.51E-74
MAGI2-AS3	-2.24574	3.430292	3.20E-73	7.95E-72
LINC00656	-3.38293	-2.36147	2.28E-71	5.47E-70
LINC01748	5.689936	1.433831	6.75E-69	1.52E-67
MIR4713HG	5.479885	-1.0195	1.75E-68	3.90E-67
LINC01633	6.915699	-1.6564	6.19E-68	1.37E-66
POU6F2-AS2	7.326181	-0.21021	1.29E-67	2.84E-66
MIR205HG	6.155437	6.7325	1.34E-67	2.93E-66
LINC02163	7.441752	-0.74387	1.43E-67	3.12E-66
LINC01980	8.862842	2.257274	1.79E-67	3.90E-66
MED4-AS1	-2.32182	-2.39428	5.43E-67	1.17E-65
LINC00491	7.296171	0.852412	1.17E-66	2.51E-65
LINC02489	-3.9096	-1.69181	2.33E-65	4.84E-64
RARA-AS1	-1.59008	1.072713	3.17E-64	6.37E-63
TMPO-AS1	2.470467	2.219839	1.54E-63	3.03E-62
LINC02428	7.028693	-0.08947	1.74E-63	3.41E-62
LINC02038	-3.12115	-1.07678	7.47E-63	1.44E-61
CALML3-AS1	6.170223	2.587598	4.31E-62	8.14E-61
VPS9D1-AS1	3.697623	3.469087	1.08E-59	1.93E-58
TFAP2A-AS1	3.359182	0.122652	1.79E-59	3.18E-58
WDFY3-AS2	-1.73709	0.981235	2.89E-59	5.11E-58
PCAT6	3.144941	2.64227	1.80E-58	3.13E-57
MGC27382	-3.94093	-0.9269	1.84E-58	3.19E-57

G2E3-AS1	8.271191	0.70698	2.30E-58	3.96E-57
DDX11-AS1	2.97314	0.358784	2.56E-58	4.41E-57
FOXD3-AS1	7.32767	0.70989	2.64E-58	4.54E-57
LINC00163	-3.65016	-2.53593	1.25E-57	2.12E-56
STARD13-AS	-2.79147	-2.70724	1.99E-57	3.35E-56
LINC01836	-2.61302	-0.37001	9.29E-57	1.55E-55
OGFRP1	2.625772	0.564856	1.64E-56	2.70E-55
CARMN	-1.99386	0.292934	3.99E-56	6.49E-55
VIPR1-AS1	-2.42475	-0.67853	6.62E-56	1.06E-54
HHIP-AS1	-2.69834	1.021246	2.46E-55	3.88E-54
PAXIP1-AS2	-1.32884	2.016748	7.85E-55	1.23E-53
LINC01863	-4.37529	-2.81616	8.37E-55	1.31E-53
DLX6-AS1	7.252354	2.96411	3.36E-54	5.14E-53
ADAMTSL4-AS1	-2.03847	-0.98071	4.66E-54	7.11E-53
LINC02185	-3.1494	-1.21583	2.29E-53	3.42E-52
BCRP3	-2.30613	-0.30552	3.03E-53	4.52E-52
ZEB2-AS1	-2.03827	-1.97446	3.03E-52	4.41E-51
HOXC13-AS	8.036722	0.678788	2.81E-51	3.99E-50
LINC00519	5.976881	2.264552	1.27E-50	1.77E-49
SNHG1	2.057868	5.987783	1.94E-50	2.69E-49
LINC00315	-1.98548	-2.95448	2.79E-50	3.86E-49
CADM3-AS1	-3.17379	-0.99645	4.17E-50	5.72E-49
APOA1-AS	-1.93662	-2.99608	4.86E-50	6.65E-49
MELTF-AS1	3.045711	2.463466	1.25E-49	1.70E-48
SLC2A1-AS1	3.395883	1.174252	2.33E-49	3.14E-48
PVT1	2.46946	4.097362	6.63E-49	8.85E-48
LINC00958	5.772011	4.332938	6.77E-48	8.70E-47
LINC02471	-3.56789	-0.73662	3.37E-47	4.22E-46
LINC01105	-3.81981	-1.97035	2.11E-46	2.56E-45
LINC00924	-2.38395	-1.2037	4.51E-46	5.42E-45
DARS-AS1	2.196727	1.460484	5.24E-46	6.28E-45
HAGLROS	4.465841	1.393178	9.93E-46	1.18E-44
PCAT7	4.045035	0.752398	3.53E-45	4.09E-44
MAMDC2-AS1	-1.91081	-1.04668	3.53E-45	4.09E-44
LINC00472	-2.17949	-0.50166	4.04E-45	4.68E-44
NPSR1-AS1	6.582247	-0.97531	4.43E-45	5.11E-44
LINC02104	-2.48011	-2.88576	6.46E-45	7.40E-44
SFTPD-AS1	-2.60442	-2.96711	3.68E-44	4.10E-43
LINC00982	-2.58856	-0.04826	3.95E-44	4.40E-43
LINC01096	6.201861	-1.01258	5.17E-44	5.74E-43
LINC00668	7.197749	3.186933	1.21E-43	1.33E-42
HOXC-AS2	5.747651	-0.00922	2.48E-43	2.69E-42
CASC8	4.763662	1.296929	3.07E-43	3.32E-42
MIR2052HG	4.875246	-0.49233	3.12E-43	3.37E-42
PARAL1	-3.54286	-0.0165	3.39E-43	3.65E-42
MEF2C-AS1	-2.21859	-1.65431	8.73E-43	9.29E-42
SNHG4	2.683121	2.146419	1.13E-42	1.20E-41
TMEM220-AS1	-1.69898	-1.13156	1.32E-42	1.39E-41
BAIAP2-DT	-1.29104	4.570582	1.66E-42	1.74E-41
LINC01108	-3.21585	-1.29266	2.79E-42	2.91E-41
DSG1-AS1	6.850043	0.04806	2.90E-42	3.02E-41
CASC2	-1.48669	0.946979	4.43E-42	4.59E-41
LINC01703	3.093591	0.384759	9.92E-42	1.02E-40
LINC01967	6.181263	-1.53448	1.53E-41	1.56E-40
ELN-AS1	-2.77796	-0.47267	3.02E-41	3.06E-40
EP300-AS1	-1.6535	-0.8099	4.43E-41	4.44E-40
LINC02126	-2.46868	-2.51995	1.02E-40	1.01E-39
LINC00898	7.434235	-0.0963	1.10E-40	1.09E-39

LINC02562	3.44515	2.757765	5.30E-40	5.10E-39
LINC02289	-2.24569	-1.09176	8.66E-40	8.26E-39
IL10RB-DT	-1.27297	0.167837	3.32E-39	3.08E-38
LINC01150	-2.01968	-2.39827	7.21E-39	6.62E-38
LINC01873	4.949645	-2.01318	2.98E-38	2.67E-37
KC6	5.042917	2.131069	3.68E-38	3.28E-37
LINC01807	7.247501	-0.01164	4.05E-38	3.59E-37
LINC02285	-1.91477	-1.96504	5.62E-38	4.95E-37
LINC00551	-2.61053	-1.39038	1.17E-37	1.03E-36
FEZF1-AS1	5.978581	2.86126	1.18E-37	1.03E-36
LYRM4-AS1	1.652675	1.12701	1.74E-37	1.52E-36
HOXA11-AS	6.017133	0.362265	1.89E-37	1.65E-36
GHET1	2.097567	-0.14206	2.40E-37	2.08E-36
MYO16-AS1	-3.04106	-1.08392	3.02E-37	2.61E-36
LINC02466	6.346282	-1.44695	3.33E-37	2.87E-36
DUXAP8	3.470342	1.755463	4.57E-37	3.92E-36
TRPM2-AS	4.718004	1.505117	4.70E-37	4.03E-36
LINC00261	-3.44601	2.226233	5.56E-37	4.76E-36
FIRRE	3.815587	1.314255	8.52E-37	7.22E-36
LMO7DN	-2.40251	-2.95636	9.45E-37	8.00E-36
PTCSC3	-2.59206	-2.29274	6.13E-36	5.05E-35
LINC02018	-1.73191	-2.1519	1.18E-35	9.61E-35
C8orf34-AS1	-2.98589	1.677769	1.35E-35	1.10E-34
SOX21-AS1	4.148084	3.314629	1.48E-35	1.20E-34
AGAP1-IT1	-1.99413	-1.95778	1.58E-35	1.27E-34
KTN1-AS1	1.832634	1.973073	3.65E-35	2.90E-34
LINC01234	7.150922	2.610968	3.91E-35	3.11E-34
CYP1B1-AS1	-1.79895	-0.17741	4.27E-35	3.38E-34
LINC01305	6.805632	0.136971	6.50E-35	5.11E-34
LINC02595	3.419703	-1.09117	6.84E-35	5.37E-34
DEPDC1-AS1	3.559928	-2.76232	6.88E-35	5.40E-34
KDM4A-AS1	2.748645	1.5281	1.09E-34	8.48E-34
CYP4A22-AS1	3.038142	-1.0963	2.57E-34	1.97E-33
LINC01698	6.795296	-1.94431	9.40E-34	7.07E-33
LINC00641	-1.39079	2.767381	9.51E-34	7.15E-33
C5orf66-AS1	7.809083	0.789907	1.04E-33	7.79E-33
ZFPM2-AS1	5.218011	2.141874	1.07E-33	8.01E-33
MIR29B2CHG	-2.00803	1.915799	1.16E-33	8.70E-33
FRMD6-AS1	2.432893	-0.01623	1.44E-33	1.07E-32
LINC00640	3.605278	0.36563	1.68E-33	1.25E-32
FAM83A-AS1	6.34548	1.563837	2.22E-33	1.64E-32
LINC02265	-2.45606	-2.59986	2.27E-33	1.68E-32
TARID	-2.5455	-0.23669	2.43E-33	1.79E-32
HOXA10-AS	5.601971	-0.9305	2.64E-33	1.94E-32
LINC01451	4.270417	2.198405	2.91E-33	2.13E-32
MIR2117HG	5.521074	-0.6552	3.26E-33	2.38E-32
LINC00887	4.054799	-0.52157	4.41E-33	3.20E-32
LINC02253	6.582203	1.046001	8.48E-33	6.11E-32
LINC02156	3.611326	-2.86246	1.15E-32	8.20E-32
FLJ46906	2.195015	2.131255	2.14E-32	1.51E-31
LINC01977	3.940716	-0.74491	2.30E-32	1.62E-31
RUNDC3A-AS1	3.333026	0.580371	2.75E-32	1.93E-31
LINC01752	4.263631	0.238269	3.61E-32	2.52E-31
LINC01605	4.401026	1.993319	5.27E-32	3.65E-31
LINC02202	-1.60308	-1.51089	5.51E-32	3.81E-31
LINC01981	5.341213	-2.83548	5.77E-32	3.99E-31
NR2F2-AS1	-1.67365	-0.03489	1.06E-31	7.25E-31
SLC12A9-AS1	2.450295	-0.73054	1.28E-31	8.72E-31

LINC01624	-2.29579	-2.71461	1.97E-31	1.33E-30
SENCR	-1.72374	-0.4925	2.78E-31	1.86E-30
TTC39A-AS1	-2.1648	-1.80884	3.25E-31	2.17E-30
CASC19	6.620338	0.476923	3.46E-31	2.31E-30
UMODL1-AS1	-3.0485	-1.26704	3.92E-31	2.60E-30
LUARIS	4.665754	-1.08803	7.75E-31	5.08E-30
CCDC13-AS1	-1.80874	-2.47326	9.71E-31	6.33E-30
BARX1-DT	7.076152	-1.33751	1.77E-30	1.14E-29
LOXL1-AS1	1.886171	3.120049	2.36E-30	1.52E-29
LINC02541	3.221443	2.221964	3.01E-30	1.93E-29
LINC00626	7.710234	1.313773	3.27E-30	2.09E-29
LINC01503	1.992978	3.959039	3.72E-30	2.37E-29
EPB41L4A-DT	-1.30899	-0.41619	4.11E-30	2.62E-29
SNHG17	1.539481	5.11731	4.39E-30	2.79E-29
DGUOK-AS1	1.986491	1.730826	4.55E-30	2.89E-29
NAV2-AS2	-2.52279	-2.67692	5.14E-30	3.26E-29
WWC2-AS2	-1.61761	-1.14889	9.92E-30	6.19E-29
LSAMP-AS1	4.843527	-1.1727	1.14E-29	7.10E-29
LNCAROD	7.431809	0.314548	1.37E-29	8.49E-29
MIR210HG	1.989911	3.539673	1.87E-29	1.15E-28
MYOSLID	3.324541	1.206515	2.65E-29	1.62E-28
LINC01063	2.653602	-0.69226	3.74E-29	2.27E-28
LINC01506	-2.29532	-2.47244	4.92E-29	2.98E-28
C10orf25	-1.39571	1.198973	6.40E-29	3.84E-28
LINC00355	7.670274	-0.05503	6.73E-29	4.03E-28
LINC02043	4.615957	-0.94993	6.93E-29	4.14E-28
SRGAP3-AS2	-3.41884	0.799551	7.03E-29	4.21E-28
ST3GAL5-AS1	-1.83036	-2.38508	8.40E-29	5.00E-28
SATB2-AS1	3.37004	-1.77343	9.63E-29	5.73E-28
HOTTIP	6.067607	-1.87581	1.13E-28	6.69E-28
MIR9-3HG	3.367283	2.955744	1.21E-28	7.13E-28
C5orf56	-1.36451	2.460019	2.47E-28	1.45E-27
ATP2A1-AS1	2.353913	-0.26833	3.25E-28	1.89E-27
C2orf48	2.966613	-0.16866	3.64E-28	2.11E-27
LINC00996	-1.75772	-0.22634	4.22E-28	2.44E-27
LEF1-AS1	2.106732	-0.86877	4.49E-28	2.59E-27
NAALADL2-AS2	5.95139	-0.48493	5.01E-28	2.88E-27
PWAR6	-1.68335	1.187914	5.04E-28	2.90E-27
LNCOC1	2.751307	0.175159	5.95E-28	3.42E-27
PSMG3-AS1	-1.35606	2.489411	6.77E-28	3.88E-27
LINC01765	-3.30736	-2.05423	1.05E-27	5.97E-27
LINC01564	4.114247	0.881424	1.16E-27	6.58E-27
HOXC-AS1	4.136955	-0.95457	1.64E-27	9.20E-27
FLJ34503	-2.39888	-2.64172	1.65E-27	9.30E-27
LINC01711	3.54629	-1.64206	1.80E-27	1.01E-26
LINC00654	-1.49669	1.228835	2.86E-27	1.59E-26
SEMA3B-AS1	-1.68759	-1.56337	3.10E-27	1.72E-26
LINC00892	-2.05492	-1.30051	3.48E-27	1.93E-26
C10orf91	3.167378	0.751003	3.95E-27	2.18E-26
LINC00858	4.53001	-0.85971	4.17E-27	2.30E-26
RMST	-3.29045	-2.0989	4.26E-27	2.35E-26
LINC01208	5.254181	-2.50941	4.27E-27	2.35E-26
CFLAR-AS1	-1.4737	-0.43261	4.53E-27	2.49E-26
DLX2-DT	5.412974	-2.76423	7.35E-27	4.01E-26
LINC01385	5.522929	-2.58536	9.48E-27	5.13E-26
LINC01968	3.515872	-2.5559	1.27E-26	6.85E-26
LINC01249	8.357502	0.48376	1.40E-26	7.55E-26
SREBF2-AS1	1.27933	2.577885	1.57E-26	8.44E-26

PITPNA-AS1	1.377623	1.893382	1.61E-26	8.62E-26
ABCA9-AS1	4.519095	-0.24332	1.70E-26	9.09E-26
FAM83C-AS1	2.764629	-2.62308	2.12E-26	1.13E-25
SOX2-OT	5.539647	4.649621	2.40E-26	1.27E-25
LINC00592	3.011478	-1.09175	3.29E-26	1.74E-25
HAGLR	-1.57692	4.319854	4.41E-26	2.31E-25
MAGEA10-MAGEA	5.431136	-1.9976	5.06E-26	2.64E-25
ADPGK-AS1	-1.36267	-1.88664	5.79E-26	3.02E-25
LINC01206	9.433899	5.121557	6.00E-26	3.12E-25
AGAP2-AS1	1.494461	3.895077	6.44E-26	3.34E-25
ARNTL2-AS1	4.02093	-2.75231	6.51E-26	3.38E-25
SH3PXD2A-AS1	3.828419	2.164548	9.90E-26	5.09E-25
MAFG-DT	1.733784	3.0238	1.14E-25	5.86E-25
MAGEA4-AS1	8.155701	1.134907	1.27E-25	6.48E-25
SLC16A1-AS1	1.462176	2.134553	1.43E-25	7.31E-25
LINC01524	4.756324	-1.82259	2.12E-25	1.07E-24
LINC02321	2.931205	-1.54928	2.39E-25	1.21E-24
LINC01671	-2.05771	0.517566	2.45E-25	1.24E-24
LINC02457	6.177143	-2.14651	2.50E-25	1.26E-24
SATB1-AS1	2.292851	1.152254	2.60E-25	1.31E-24
LINC00393	7.838964	-0.15318	2.63E-25	1.33E-24
LINC01518	8.308274	-0.38799	3.18E-25	1.60E-24
LINC02166	-1.42817	-1.20934	5.19E-25	2.59E-24
GCC2-AS1	1.464531	-1.41014	7.14E-25	3.54E-24
GACAT2	4.224836	-2.44523	1.08E-24	5.30E-24
SLC9A3-AS1	2.814315	4.505404	1.15E-24	5.66E-24
MAFA-AS1	5.051741	-1.09119	1.38E-24	6.75E-24
HOXD-AS2	3.799673	-0.44182	1.70E-24	8.31E-24
LINC01456	6.378109	-0.86565	1.74E-24	8.48E-24
LINC02323	3.190606	-0.39724	1.80E-24	8.80E-24
LINC02542	2.394614	1.53186	2.26E-24	1.10E-23
LINC02473	4.794725	-2.68888	2.81E-24	1.36E-23
DANCR	1.89299	5.694123	3.06E-24	1.48E-23
LINC01705	3.662731	-0.97986	3.78E-24	1.82E-23
LINC02416	2.582998	-2.31441	4.67E-24	2.23E-23
LINC00941	3.761251	1.144233	5.64E-24	2.69E-23
HOTAIR	6.713693	0.502639	6.11E-24	2.91E-23
LINC01559	5.990928	1.699726	6.34E-24	3.01E-23
DLEU1	1.185427	3.007395	6.65E-24	3.16E-23
LINC00885	2.799457	2.740436	6.94E-24	3.30E-23
LINC01311	1.961551	-0.04954	6.99E-24	3.32E-23
LINC02012	3.148187	0.542421	7.17E-24	3.40E-23
PKP4-AS1	2.994438	1.024554	7.33E-24	3.47E-23
LINC01612	-2.73701	-1.7778	7.68E-24	3.64E-23
LINC01905	3.77892	-1.55013	7.73E-24	3.66E-23
LINC01561	3.778647	-1.65716	7.78E-24	3.68E-23
YEATS2-AS1	2.018413	1.4812	7.79E-24	3.69E-23
LINC00337	2.610213	-0.82986	8.72E-24	4.11E-23
LINC01012	2.099411	-0.20439	1.16E-23	5.45E-23
LINC00515	-1.65107	-3.02889	1.26E-23	5.88E-23
HOXC-AS3	6.685607	-1.02652	1.30E-23	6.06E-23
CHKB-DT	1.527264	0.131569	1.38E-23	6.44E-23
DDN-AS1	1.793608	-0.60648	1.48E-23	6.91E-23
NCK1-DT	1.466266	2.8155	1.64E-23	7.62E-23
IGFL2-AS1	5.59343	2.577192	1.74E-23	8.08E-23
LINC01322	4.498104	-1.22393	1.87E-23	8.65E-23
KIF26B-AS1	-2.25767	-2.93295	2.18E-23	1.01E-22
LINC02014	2.656471	-0.53443	3.48E-23	1.60E-22

LINC02109	5.726338	-0.94914	3.77E-23	1.72E-22
LINC01232	1.549353	2.548451	3.91E-23	1.79E-22
LINC01513	-2.55548	-2.9602	4.34E-23	1.98E-22
GAPLINC	-1.54005	-0.18582	5.43E-23	2.47E-22
LINC01615	3.450453	0.392123	5.92E-23	2.68E-22
LINC01775	2.470827	-2.91153	5.93E-23	2.68E-22
FSIP2-AS1	2.919074	-1.61247	7.04E-23	3.17E-22
LINC02598	3.672599	-1.73605	7.15E-23	3.22E-22
ZNF582-AS1	-1.4856	-0.17235	7.17E-23	3.23E-22
VPS33B-DT	1.689839	-1.36226	7.38E-23	3.32E-22
NKX2-1-AS1	-2.52838	0.255941	7.78E-23	3.49E-22
LINC00852	-1.08705	-0.74482	1.05E-22	4.69E-22
LINC01901	4.908092	-0.54783	1.23E-22	5.47E-22
MNX1-AS1	4.852627	0.314777	1.51E-22	6.68E-22
FLJ31104	-1.31137	-2.01142	1.57E-22	6.96E-22
TMEM44-AS1	1.607131	3.240726	1.71E-22	7.57E-22
SMILR	3.837384	-1.75111	1.85E-22	8.15E-22
LINC01876	2.440088	0.486974	2.13E-22	9.37E-22
LINC00942	6.986335	3.880547	2.26E-22	9.92E-22
LINC01833	6.932341	0.344285	3.12E-22	1.36E-21
LINC01558	-1.74493	-1.4225	3.13E-22	1.36E-21
CASC20	5.160712	-0.41698	4.55E-22	1.97E-21
LINC01611	6.164791	-1.71098	6.46E-22	2.77E-21
SBF2-AS1	1.54709	2.648053	7.00E-22	2.99E-21
USP30-AS1	-1.53264	0.16176	7.40E-22	3.16E-21
LINC00930	-2.47946	-1.47776	1.19E-21	5.02E-21
LINC01133	4.247515	4.170694	1.19E-21	5.03E-21
LINC01971	3.497684	-2.71092	1.24E-21	5.26E-21
LINC01393	2.226691	-1.3082	1.32E-21	5.54E-21
ALMS1-IT1	1.653699	0.684195	1.39E-21	5.83E-21
NUP50-DT	1.501793	3.662663	1.42E-21	5.98E-21
BLACAT1	2.827125	2.182076	1.49E-21	6.28E-21
SUCLG2-AS1	-1.07289	-0.80225	1.59E-21	6.66E-21
LINC00871	5.479898	-0.80118	1.77E-21	7.41E-21
TMEM147-AS1	1.680484	3.57695	1.90E-21	7.97E-21
PART1	4.4967	2.283728	1.93E-21	8.07E-21
SNHG21	1.278693	0.752077	1.95E-21	8.14E-21
CYP4F26P	3.470633	0.080844	2.05E-21	8.55E-21
LINC02345	-1.79111	0.733396	2.16E-21	9.00E-21
PARD3-AS1	2.517079	-1.18051	2.40E-21	9.99E-21
IL20RB-AS1	4.823465	-2.72495	2.41E-21	1.00E-20
LINC00630	1.10681	1.543506	3.02E-21	1.25E-20
LINC01842	4.065155	-0.68511	3.43E-21	1.42E-20
TBX2-AS1	-1.60175	0.702476	3.89E-21	1.60E-20
LINC01277	-1.48547	-2.34681	4.39E-21	1.80E-20
LINC01391	5.793976	-1.96602	4.40E-21	1.80E-20
AGAP11	-1.80916	-2.50697	4.95E-21	2.03E-20
LINC01116	2.847346	2.019356	5.70E-21	2.33E-20
UBAC2-AS1	1.443643	0.613814	6.31E-21	2.57E-20
LINC02438	5.687417	-2.43903	6.71E-21	2.73E-20
LINC01844	-2.09506	-2.97285	6.79E-21	2.76E-20
LUNAR1	2.818312	-1.78044	7.62E-21	3.09E-20
LINC02076	2.997827	-2.10001	8.47E-21	3.43E-20
UCA1	4.896021	1.934065	9.68E-21	3.91E-20
FOXD2-AS1	1.374531	2.049625	9.71E-21	3.92E-20
LINC02041	2.676261	0.269813	1.02E-20	4.10E-20
PRC1-AS1	1.760282	-1.85232	1.34E-20	5.37E-20
DCST1-AS1	1.446258	0.399995	1.41E-20	5.64E-20

LINC01460	3.058376	-0.29033	1.65E-20	6.61E-20
LINC01569	1.247774	1.604584	1.68E-20	6.72E-20
LMCD1-AS1	1.632077	0.435957	1.81E-20	7.20E-20
LINC01361	2.958715	-2.53943	2.05E-20	8.12E-20
LINC00466	4.457129	-2.85131	2.11E-20	8.38E-20
LINC02315	3.890586	0.733151	2.45E-20	9.68E-20
MLIP-IT1	3.451096	-2.38858	2.51E-20	9.88E-20
PTCSC2	4.492195	-1.87928	3.02E-20	1.19E-19
LINC01202	5.722042	-2.65026	3.03E-20	1.19E-19
LINC01614	2.854258	1.033198	3.07E-20	1.21E-19
LINC02560	2.436813	0.106861	4.86E-20	1.89E-19
STRA6LP	-1.48184	-2.1063	5.53E-20	2.15E-19
LINC01136	1.926297	-1.35001	6.59E-20	2.55E-19
RNF139-AS1	1.353752	1.181551	7.58E-20	2.93E-19
LINC00615	6.824741	-1.43174	8.71E-20	3.36E-19
LINC01266	-1.71878	-1.94348	9.08E-20	3.50E-19
ERVE-1	-1.56965	-0.09054	9.18E-20	3.54E-19
SH3BP5-AS1	-1.18582	2.65052	9.85E-20	3.79E-19
LINC01504	-1.51954	0.18152	1.02E-19	3.92E-19
MINCR	1.373195	2.151889	1.13E-19	4.32E-19
ZNF503-AS2	1.309645	2.545324	1.25E-19	4.77E-19
KCNH1-IT1	5.193381	-2.46651	1.38E-19	5.26E-19
SNHG3	1.346162	4.912214	1.45E-19	5.53E-19
ZNF252P-AS1	1.690179	-1.20596	1.50E-19	5.73E-19
LINC002481	-1.26363	-0.80737	2.12E-19	8.02E-19
GTF3C2-AS1	1.405108	-0.90568	2.18E-19	8.26E-19
PSMD6-AS2	-1.20179	-1.15709	2.64E-19	9.96E-19
STEAP2-AS1	2.841269	-2.80723	3.22E-19	1.21E-18
LINC01176	1.628284	1.169418	3.54E-19	1.33E-18
LINC01527	5.112757	-0.52331	3.81E-19	1.43E-18
LINC00896	2.49489	-1.24547	4.60E-19	1.71E-18
LINC01992	7.032721	-0.72803	5.27E-19	1.95E-18
LINC02159	3.513915	0.493445	5.49E-19	2.03E-18
LINC02561	3.777207	0.830832	5.62E-19	2.08E-18
MIR1-1HG-AS1	-1.96293	-2.89832	5.87E-19	2.17E-18
LINC01607	1.994503	-0.54951	6.12E-19	2.26E-18
LINC01297	6.296994	-2.12168	8.05E-19	2.95E-18
LINC02178	6.348736	-1.03146	8.30E-19	3.04E-18
LINC00649	1.283841	3.537028	8.44E-19	3.09E-18
LINC02544	2.583563	-0.94474	8.54E-19	3.13E-18
ELF3-AS1	1.360098	1.160318	8.66E-19	3.17E-18
HOXB-AS4	5.115925	-1.47702	9.13E-19	3.34E-18
DIRC3	3.139197	1.775647	9.20E-19	3.37E-18
LINC02310	4.47273	-2.66656	9.82E-19	3.59E-18
LINC00461	5.294827	-1.19601	1.02E-18	3.74E-18
ACBD3-AS1	-1.40763	-0.43335	1.15E-18	4.17E-18
LINC00392	9.429893	1.280993	1.16E-18	4.23E-18
SALRNA1	2.409234	-2.13335	1.40E-18	5.07E-18
LINC00470	4.241554	1.008133	1.75E-18	6.31E-18
LINC01963	-1.12239	2.286442	1.95E-18	7.02E-18
SNHG25	1.719178	-0.49361	2.44E-18	8.73E-18
NCBP2-AS1	1.806294	-1.21466	2.60E-18	9.28E-18
LINC00462	5.182907	-1.00912	2.95E-18	1.05E-17
TAF1A-AS1	1.206853	0.721709	3.16E-18	1.12E-17
HCG15	1.714935	-0.45888	3.84E-18	1.36E-17
XXYLT1-AS1	2.932204	-2.67813	3.97E-18	1.41E-17
HCG25	1.115468	-0.47208	4.05E-18	1.43E-17
LINC00536	5.788357	-1.87431	4.57E-18	1.61E-17

SH3RF3-AS1	-1.20719	-0.30035	4.86E-18	1.71E-17
VIM-AS1	-1.28549	1.247826	5.05E-18	1.78E-17
LINC01592	3.83023	-2.54855	5.21E-18	1.83E-17
LINC00115	1.291925	-0.20741	5.54E-18	1.95E-17
LINP1	4.169943	0.317439	5.68E-18	1.99E-17
APCDD1L-DT	3.873074	-0.05688	5.83E-18	2.04E-17
LINC01915	-1.71761	-2.31203	7.24E-18	2.53E-17
MAPK6-DT	1.948357	-2.13008	7.69E-18	2.68E-17
IGF2BP2-AS1	3.217613	-1.36217	8.18E-18	2.85E-17
LINC01117	2.540376	-1.45809	8.21E-18	2.86E-17
FLJ46284	-1.33295	-1.39186	8.41E-18	2.92E-17
LINC02137	3.525977	-0.99816	1.10E-17	3.78E-17
CARD8-AS1	-1.05274	1.789086	1.32E-17	4.54E-17
TM4SF19-AS1	1.913518	0.835592	1.46E-17	5.00E-17
PCAT1	2.069205	0.099795	1.47E-17	5.04E-17
RBMS3-AS3	-1.62036	-1.87186	1.59E-17	5.43E-17
DTX2P1-UPK3BP1	1.578868	0.93715	1.60E-17	5.44E-17
DLEU2	1.147297	2.53126	1.76E-17	5.97E-17
ATP6V1B1-AS1	3.068736	-2.56813	1.92E-17	6.50E-17
LINC00173	2.321121	0.495182	1.92E-17	6.52E-17
LINC00920	-1.3784	0.161391	1.95E-17	6.62E-17
DLG1-AS1	2.03433	-0.82426	1.96E-17	6.65E-17
NEXN-AS1	-1.21656	-2.36145	2.31E-17	7.81E-17
LINC00665	2.246212	4.569685	2.34E-17	7.92E-17
MIR31HG	4.146423	-0.24387	2.43E-17	8.19E-17
DLEU7-AS1	1.65078	-1.77064	2.53E-17	8.53E-17
TM4SF1-AS1	2.688406	0.427355	2.60E-17	8.76E-17
LINC01013	-1.84339	-2.5366	2.76E-17	9.28E-17
NFIA-AS2	-1.73669	-2.55579	3.24E-17	1.09E-16
LINC01697	4.5988	0.471454	3.91E-17	1.31E-16
MIR137HG	6.221605	-0.87081	3.99E-17	1.33E-16
MGC32805	-1.86725	0.237883	4.03E-17	1.35E-16
DSCR9	2.341099	-1.61723	4.06E-17	1.35E-16
C5orf66	1.702253	1.075819	4.68E-17	1.56E-16
WNT5A-AS1	2.262095	0.688379	4.84E-17	1.61E-16
CASC11	3.061606	-1.34571	5.21E-17	1.73E-16
TBL1XR1-AS1	2.59941	-2.85882	5.54E-17	1.84E-16
GDNF-AS1	3.799153	0.036126	6.21E-17	2.06E-16
KLHL7-DT	1.869418	0.375443	7.48E-17	2.47E-16
LINC02141	5.347756	-2.71088	8.13E-17	2.67E-16
C21orf62-AS1	-1.04382	-0.64711	8.24E-17	2.71E-16
LINC01146	-1.52746	-1.89491	8.89E-17	2.92E-16
AFAP1-AS1	4.628659	4.170044	1.08E-16	3.52E-16
LINC01194	8.007494	-0.03644	1.19E-16	3.87E-16
ELDR	4.976869	-0.87823	1.37E-16	4.45E-16
DGCR9	2.27233	0.133332	1.39E-16	4.50E-16
SUCLA2-AS1	1.312109	-1.47562	1.40E-16	4.56E-16
C2-AS1	-1.45521	-2.74973	1.42E-16	4.62E-16
GTSE1-DT	1.58956	0.121306	1.46E-16	4.75E-16
FLJ42969	2.635666	-1.76249	1.49E-16	4.82E-16
PICART1	-1.41045	-0.07789	1.63E-16	5.26E-16
LINC01571	-2.89728	-2.85737	1.63E-16	5.26E-16
C1orf220	1.838168	-0.12472	1.64E-16	5.29E-16
F11-AS1	-2.29886	-1.4333	1.64E-16	5.30E-16
SLC12A5-AS1	2.211282	-0.69125	1.74E-16	5.60E-16
LINC01267	-1.72221	-1.58984	1.86E-16	5.98E-16
TEX41	2.254809	1.355491	2.23E-16	7.13E-16
LINC02527	3.894647	-1.74113	2.71E-16	8.66E-16

STK4-AS1	1.348632	-0.81708	3.09E-16	9.81E-16
LINC01629	4.536384	-0.53923	3.52E-16	1.12E-15
PTGES2-AS1	2.159638	-2.0213	3.84E-16	1.22E-15
PRRT3-AS1	1.555563	0.390933	3.85E-16	1.22E-15
LNCTAM34A	1.265225	1.068103	4.65E-16	1.47E-15
LINC00908	-1.46968	-0.42738	4.73E-16	1.49E-15
LINC01956	5.225683	-1.62098	4.95E-16	1.56E-15
LINC01869	1.69615	-2.07433	5.98E-16	1.87E-15
LINC02081	2.346299	0.117412	6.03E-16	1.89E-15
CAPN10-DT	1.155016	1.273381	6.97E-16	2.17E-15
LINC00184	2.665381	-1.67135	7.11E-16	2.22E-15
ZFHX4-AS1	5.433625	-0.95899	7.82E-16	2.43E-15
RNF217-AS1	1.844388	0.583935	8.58E-16	2.66E-15
LINC02086	3.178653	0.113708	9.71E-16	3.00E-15
ELFN1-AS1	3.860332	-0.2256	1.36E-15	4.16E-15
ZKSCAN2-DT	1.526277	1.256725	1.42E-15	4.34E-15
MIR548XHG	7.743794	-0.31676	1.55E-15	4.72E-15
LINC00992	2.743792	0.192737	1.85E-15	5.62E-15
LINC00310	-1.05763	-1.29129	1.87E-15	5.67E-15
LINC00520	3.389722	0.431764	2.15E-15	6.51E-15
LINC01583	3.222289	-2.18182	2.19E-15	6.61E-15
LINC02256	-1.07214	-1.7027	2.40E-15	7.23E-15
LINC00449	1.571932	-2.64307	2.41E-15	7.28E-15
SNHG10	1.136964	2.310816	2.47E-15	7.43E-15
LINC02588	5.162806	-1.56677	2.62E-15	7.87E-15
STEAP3-AS1	2.197652	-0.12581	2.64E-15	7.92E-15
WASIR2	2.571605	-1.69406	2.64E-15	7.92E-15
HLA-DQB1-AS1	-1.45462	0.356725	2.79E-15	8.39E-15
ZNF436-AS1	1.109276	1.522318	3.37E-15	1.01E-14
LINC00518	4.975425	-2.33762	3.54E-15	1.06E-14
UBE2Q1-AS1	1.478319	-1.91483	4.20E-15	1.25E-14
LINC01419	9.151155	1.064245	4.26E-15	1.27E-14
CLEC12A-AS1	3.238891	-1.21543	4.43E-15	1.32E-14
LINC00535	-1.3913	-1.61868	4.44E-15	1.32E-14
LINC01994	4.258848	-2.81767	5.96E-15	1.76E-14
LINC01214	4.336357	-2.8539	6.85E-15	2.02E-14
SIAH2-AS1	1.883651	-1.95599	7.70E-15	2.26E-14
VAC14-AS1	1.453768	-1.60463	7.88E-15	2.31E-14
LINC02387	3.211895	-2.43877	1.10E-14	3.20E-14
SAMD12-AS1	2.274662	0.070055	1.21E-14	3.51E-14
KCTD21-AS1	1.485057	1.267862	1.25E-14	3.63E-14
LINC02393	6.31123	-0.96599	1.39E-14	4.03E-14
LINC00862	2.147291	-2.02365	1.48E-14	4.30E-14
FAM222A-AS1	2.409149	-1.43343	1.54E-14	4.44E-14
LINC01854	7.18356	-0.91263	1.78E-14	5.14E-14
ATP13A4-AS1	-2.50446	-1.06261	2.01E-14	5.78E-14
FLJ12825	2.126141	-1.50649	2.08E-14	5.97E-14
LY86-AS1	-1.66688	-2.73726	2.09E-14	6.02E-14
MBNL1-AS1	-1.06173	2.499605	2.14E-14	6.16E-14
OVOL1-AS1	2.418523	-2.67125	2.18E-14	6.27E-14
MIS18A-AS1	1.39298	-2.04449	2.21E-14	6.35E-14
LINC00629	2.142721	-2.70923	2.28E-14	6.55E-14
LINC02377	7.389384	-1.40811	2.59E-14	7.40E-14
LINC01376	1.066848	0.879748	2.93E-14	8.37E-14
LINC02288	-1.17599	-2.45061	2.95E-14	8.40E-14
BRWD1-AS2	1.283513	-0.7085	3.02E-14	8.60E-14
LINC01287	6.529691	1.649061	3.11E-14	8.86E-14
LINC01896	7.034882	-1.17186	3.18E-14	9.04E-14

LINC01918	3.030415	-2.93318	3.66E-14	1.04E-13
LINC02068	2.394783	-0.65803	3.76E-14	1.07E-13
LINC01639	5.869125	-2.5375	3.92E-14	1.11E-13
TMEM132D-AS1	7.732009	-0.10958	4.75E-14	1.34E-13
ESRG	7.152773	2.538233	4.78E-14	1.35E-13
DLGAP1-AS2	1.638985	2.973123	4.81E-14	1.35E-13
LINC02535	3.219735	-1.53827	6.08E-14	1.70E-13
DLG5-AS1	1.181164	-0.69782	6.53E-14	1.83E-13
RFPL3S	1.557169	-0.83721	7.62E-14	2.12E-13
DNAJC9-AS1	1.079636	0.008767	7.68E-14	2.14E-13
LHX1-DT	6.057926	-1.62772	7.70E-14	2.15E-13
LINC01549	4.961526	-1.79515	7.83E-14	2.18E-13
EWSAT1	3.241616	0.211508	8.05E-14	2.24E-13
LINC02587	-1.81697	-2.82239	8.67E-14	2.41E-13
IRAIN	2.051758	-2.30125	1.04E-13	2.88E-13
RNF144A-AS1	1.812754	0.695524	1.05E-13	2.92E-13
MIR193BHG	1.742866	-0.35291	1.09E-13	3.01E-13
HORMAD2-AS1	-1.69124	-2.18258	1.13E-13	3.12E-13
FAM181A-AS1	-2.03733	-1.6165	1.30E-13	3.57E-13
SLX1A-SULT1A3	1.646855	-2.43075	1.70E-13	4.66E-13
C22orf34	-1.29031	1.22089	1.86E-13	5.07E-13
EXTL3-AS1	1.709661	-0.47152	1.88E-13	5.12E-13
LINC02208	3.640422	-2.68063	1.97E-13	5.36E-13
MANCR	2.917219	0.094556	2.08E-13	5.65E-13
STAM-AS1	1.381342	-1.12428	2.13E-13	5.80E-13
WDR86-AS1	-1.61561	1.265658	2.34E-13	6.35E-13
LINC02031	3.744251	-0.04389	2.35E-13	6.39E-13
OTX2-AS1	4.232117	-2.25606	2.40E-13	6.49E-13
LINC01127	3.480692	2.310819	2.40E-13	6.50E-13
UNC5B-AS1	2.065357	-0.01293	2.58E-13	6.99E-13
MIAT	2.312744	5.254382	2.80E-13	7.55E-13
LINC02454	3.260051	-1.59686	2.94E-13	7.94E-13
LINC00601	3.922642	-2.70997	2.97E-13	8.02E-13
LINC00504	2.758233	1.765939	3.39E-13	9.12E-13
LINC00271	-1.08236	-1.39942	3.49E-13	9.38E-13
LINC02519	-1.33139	-0.9455	3.57E-13	9.59E-13
CASK-AS1	1.662708	-2.90971	3.59E-13	9.64E-13
LINC02404	7.477097	-0.50276	3.73E-13	1.00E-12
SMAD5-AS1	1.917131	-1.99521	3.76E-13	1.01E-12
RHPN1-AS1	1.360705	1.075602	3.91E-13	1.05E-12
LINC01356	2.558867	-1.52209	4.13E-13	1.11E-12
SMC2-AS1	-1.22681	-1.30897	4.29E-13	1.15E-12
MGAT3-AS1	-1.91944	-2.83642	4.31E-13	1.15E-12
PTPRG-AS1	1.445037	0.406204	4.45E-13	1.19E-12
LHFPL3-AS1	-2.19142	-2.71987	4.52E-13	1.21E-12
LINC01687	5.426493	-2.9062	4.76E-13	1.27E-12
LINC01979	2.659966	-1.11967	5.01E-13	1.33E-12
CDKN2A-DT	3.24451	-2.76804	5.10E-13	1.36E-12
SPATA3-AS1	2.340688	-2.74754	5.33E-13	1.42E-12
MYHAS	2.1826	-2.48172	5.57E-13	1.48E-12
LINC00501	4.35765	-1.69761	6.36E-13	1.68E-12
CAMTA1-DT	1.371426	-1.87405	6.78E-13	1.79E-12
LINC01816	1.628361	-0.00058	7.28E-13	1.92E-12
LINC01914	-1.47347	-1.25854	7.30E-13	1.92E-12
LINC02158	-1.46323	-2.69525	7.67E-13	2.02E-12
LINC02362	1.862564	1.342137	8.49E-13	2.22E-12
TRG-AS1	-1.10122	1.014217	8.94E-13	2.34E-12
LINC01269	2.525484	-0.7522	9.07E-13	2.37E-12

LINC01348	1.975444	0.719428	9.22E-13	2.41E-12
RPS6KA2-IT1	-1.32473	-2.55912	9.24E-13	2.42E-12
LINC01587	3.065089	-1.82393	1.13E-12	2.94E-12
LINC00707	2.874351	-0.17529	1.21E-12	3.14E-12
LINC01521	1.217681	0.96529	1.35E-12	3.50E-12
LINC01678	-1.24022	-2.98631	1.36E-12	3.53E-12
LINC00165	-2.51055	-2.9284	1.41E-12	3.66E-12
FLJ16779	2.806113	-0.39316	1.50E-12	3.87E-12
LIVAR	2.643406	-2.29906	1.60E-12	4.13E-12
C1orf147	1.406452	-1.25735	1.65E-12	4.26E-12
LINC01091	-1.33756	-0.31745	1.70E-12	4.40E-12
CEP83-DT	1.238505	-0.59295	2.19E-12	5.59E-12
LINC02128	3.777171	-2.78954	2.44E-12	6.23E-12
LINC01964	4.672508	-2.30109	2.49E-12	6.36E-12
TNFRSF14-AS1	-1.08759	1.814326	2.61E-12	6.65E-12
C9orf163	1.383073	-1.08005	2.68E-12	6.82E-12
LINC00659	3.333888	-2.1774	2.78E-12	7.06E-12
STAG3L5P-PVRIG2	1.162759	1.676701	2.90E-12	7.36E-12
LINC01995	4.234811	-2.45121	3.07E-12	7.77E-12
MUC20-OT1	1.222142	5.067858	3.07E-12	7.78E-12
IL6R-AS1	-1.00622	-1.80987	3.45E-12	8.70E-12
TDRG1	5.339904	-2.29769	3.62E-12	9.13E-12
UCKL1-AS1	-1.104	0.468556	3.67E-12	9.25E-12
LINC01424	1.396482	-2.0366	3.75E-12	9.43E-12
PCED1B-AS1	-1.06493	3.052514	3.75E-12	9.45E-12
TCF4-AS1	3.149672	-1.80642	4.73E-12	1.19E-11
LINC01366	-1.26471	-2.69886	4.93E-12	1.23E-11
FAM230C	6.713407	-1.67853	5.62E-12	1.40E-11
OVCH1-AS1	-1.67399	-2.77708	5.76E-12	1.43E-11
LINC02100	1.701781	-2.05036	5.91E-12	1.47E-11
FLJ36000	6.575094	-1.41638	6.24E-12	1.55E-11
DNMBP-AS1	-1.05138	-1.20098	6.35E-12	1.58E-11
LINC00525	2.192346	-1.4629	6.69E-12	1.66E-11
LINC02475	3.531474	-1.26894	6.99E-12	1.73E-11
LINC01121	1.813084	-1.34199	7.19E-12	1.78E-11
SSTR5-AS1	4.135509	-1.62915	7.23E-12	1.79E-11
LINC01224	3.47005	1.470735	8.10E-12	2.00E-11
LINC01798	-1.21456	-2.69782	8.20E-12	2.02E-11
INSYN1-AS1	3.530817	-1.40272	8.37E-12	2.06E-11
STK24-AS1	1.290524	-0.92605	8.43E-12	2.08E-11
PKIA-AS1	2.019523	-2.10436	9.31E-12	2.29E-11
LINC02167	8.329695	0.066219	9.61E-12	2.36E-11
LINC00051	4.666188	-2.44094	1.05E-11	2.58E-11
LINC02476	6.211895	-2.04741	1.12E-11	2.73E-11
LINC01546	2.337494	-2.28729	1.28E-11	3.11E-11
FER1L6-AS2	5.414497	-2.11326	1.31E-11	3.21E-11
OSTM1-AS1	4.756302	-2.77375	1.34E-11	3.27E-11
LINC01910	2.999534	-2.91349	1.36E-11	3.31E-11
DIO3OS	-1.44371	0.920118	1.53E-11	3.72E-11
LINC02207	-1.35874	-2.10039	1.72E-11	4.16E-11
LEMD1-AS1	2.130746	-2.22729	1.83E-11	4.42E-11
LINC00689	-1.88041	-0.95706	2.07E-11	4.99E-11
LINC01370	7.444764	-0.76713	2.31E-11	5.56E-11
FOXCUT	2.515281	-2.28632	2.34E-11	5.64E-11
ERVMER61-1	5.921209	-2.48813	2.35E-11	5.66E-11
HOTAIRM1	1.21239	2.882381	2.46E-11	5.91E-11
LINC00484	-1.00412	-2.7906	2.53E-11	6.08E-11
CERS3-AS1	1.809328	-1.52221	2.69E-11	6.44E-11

LINC02246	1.34671	-1.69416	2.75E-11	6.59E-11
MYCNOS	3.744929	-1.97369	3.02E-11	7.20E-11
PRMT5-AS1	1.085682	-1.30062	3.37E-11	8.03E-11
LINC02582	6.3407	0.335735	3.40E-11	8.11E-11
LINC00652	1.610681	-1.58402	3.47E-11	8.26E-11
LINC01143	3.343849	-2.35164	3.56E-11	8.48E-11
RBAKDN	2.948765	-2.31861	3.60E-11	8.56E-11
HIF1A-AS1	1.761254	-1.80869	3.80E-11	9.02E-11
SIRLNT	8.802114	0.458148	4.35E-11	1.03E-10
PKN2-AS1	-1.10537	-2.63668	4.47E-11	1.05E-10
LINC01600	1.679987	-2.46205	4.66E-11	1.10E-10
LINC02223	3.559052	-2.70867	5.04E-11	1.19E-10
LINC02026	1.387493	-0.54918	5.09E-11	1.20E-10
MGC12916	1.489804	0.13768	5.10E-11	1.20E-10
LINC01213	3.373725	-2.88546	5.69E-11	1.34E-10
LINC00240	1.327078	1.206394	5.70E-11	1.34E-10
LINC01511	4.877089	-1.09555	6.66E-11	1.56E-10
LINC00174	1.090098	3.510732	7.39E-11	1.72E-10
LINC01341	1.533932	0.460566	7.72E-11	1.80E-10
SCHLAP1	5.915244	-2.53259	8.48E-11	1.97E-10
TMEM254-AS1	1.106149	1.44774	8.58E-11	1.99E-10
MYLK-AS1	1.034551	-0.12189	8.96E-11	2.08E-10
LINC02267	6.496248	-1.6469	9.51E-11	2.20E-10
SNHG26	1.21021	2.083	1.01E-10	2.34E-10
LINC01535	2.007282	-0.17159	1.05E-10	2.42E-10
HS1BP3-IT1	-1.14182	-2.65777	1.07E-10	2.46E-10
LGALS8-AS1	1.259202	-1.64172	1.07E-10	2.47E-10
LINC01336	-1.06125	-2.5914	1.21E-10	2.77E-10
THORLNC	1.609696	-1.05366	1.23E-10	2.82E-10
LINC01783	-1.61314	-2.47439	1.43E-10	3.26E-10
B3GALT5-AS1	3.188977	-0.38427	1.47E-10	3.35E-10
SNX29P2	-1.1596	-2.73681	1.49E-10	3.39E-10
LINC02551	2.406903	-1.59984	1.58E-10	3.59E-10
MSC-AS1	1.492079	3.386551	1.83E-10	4.16E-10
LINC02492	5.872351	-2.70144	2.13E-10	4.82E-10
H19	3.311202	7.187029	2.23E-10	5.05E-10
LINC02505	4.7717	-2.75284	2.25E-10	5.09E-10
LINC01303	1.806	-0.58083	2.26E-10	5.09E-10
LINC01543	3.58768	-3.00994	2.74E-10	6.14E-10
LINC01344	2.391149	-2.55812	2.86E-10	6.41E-10
STAU2-AS1	1.50839	-1.03617	2.99E-10	6.70E-10
LINC02301	4.507618	-1.86718	3.16E-10	7.06E-10
LINC00648	3.462414	0.60851	3.16E-10	7.07E-10
CYYR1-AS1	2.2942	-2.1669	3.26E-10	7.27E-10
LMF1-AS1	-1.33638	-2.25215	3.27E-10	7.29E-10
LINC01126	1.490524	-0.78556	3.28E-10	7.33E-10
LINC01050	1.941856	-2.40278	3.43E-10	7.65E-10
LINC01205	4.998728	-2.57679	3.61E-10	8.04E-10
UTAT33	1.18314	-1.71146	3.96E-10	8.79E-10
LINC00460	2.874584	0.22404	4.31E-10	9.55E-10
SNHG19	1.067636	3.2253	4.48E-10	9.93E-10
POT1-AS1	1.12485	0.411551	4.60E-10	1.02E-09
LINC02579	2.068067	-2.47728	4.95E-10	1.10E-09
DSCR8	7.127519	-0.45678	5.45E-10	1.20E-09
RN7SL832P	1.242215	-0.32717	5.53E-10	1.22E-09
LINC00471	1.025509	-0.8577	6.63E-10	1.46E-09
LINC01355	1.124213	2.2858	7.70E-10	1.69E-09
LINC01139	2.927205	1.893502	8.11E-10	1.77E-09

LINC02484	5.893789	-2.58678	8.37E-10	1.83E-09
LINC01667	5.979938	-0.75194	9.15E-10	1.99E-09
ITGB1-DT	1.71501	-2.14756	1.23E-09	2.66E-09
LINC01395	2.769156	-2.91181	1.25E-09	2.70E-09
LINC01970	1.307404	-2.30243	1.32E-09	2.85E-09
LINC01801	-1.16571	-0.97553	1.61E-09	3.46E-09
LINC01359	-1.0065	-1.614	1.73E-09	3.71E-09
LINC01929	2.109472	0.445502	1.75E-09	3.74E-09
TNKS2-AS1	1.173233	-0.83714	1.88E-09	4.02E-09
PIK3CD-AS2	1.099653	1.02407	1.91E-09	4.07E-09
LINC01254	3.457525	-2.71762	2.00E-09	4.27E-09
MDS2	-1.06439	-2.27455	2.06E-09	4.40E-09
LINC02525	6.125587	-1.28382	2.57E-09	5.46E-09
CSTF3-DT	1.268356	-2.55797	2.76E-09	5.84E-09
LINC01608	5.937454	-2.54268	2.82E-09	5.98E-09
SLC25A25-AS1	1.087938	3.205822	2.86E-09	6.04E-09
DSG2-AS1	1.16485	-0.73977	3.05E-09	6.45E-09
MPPED2-AS1	3.335189	-2.71034	3.08E-09	6.50E-09
LINC02005	3.071909	-2.4883	3.18E-09	6.70E-09
ADARB2-AS1	2.781872	-2.40554	3.31E-09	6.97E-09
LINC01160	2.133228	0.137511	4.37E-09	9.14E-09
SLC7A11-AS1	2.339882	-0.27409	4.53E-09	9.46E-09
DKFZp779M0652	-1.16117	-2.06659	5.20E-09	1.08E-08
MMP2-AS1	-1.31484	-0.2418	5.64E-09	1.17E-08
FGF12-AS2	2.422792	-3.03845	5.77E-09	1.20E-08
HDHD5-AS1	1.114624	-1.86375	5.89E-09	1.22E-08
LINC01087	3.864289	-2.36987	6.66E-09	1.38E-08
HPN-AS1	1.714392	-1.35138	7.07E-09	1.46E-08
MCCC1-AS1	1.319099	0.26485	7.25E-09	1.49E-08
LINC01426	1.453064	1.554908	7.56E-09	1.56E-08
LINC01193	5.523343	-2.75697	7.75E-09	1.59E-08
DENND5B-AS1	1.771123	-2.61291	1.00E-08	2.05E-08
EIPR1-IT1	1.541547	-2.11954	1.03E-08	2.10E-08
KRTAP5-AS1	-1.25733	-1.35911	1.04E-08	2.12E-08
BCAR4	4.139541	-2.36118	1.12E-08	2.27E-08
KIRREL3-AS1	3.851129	-2.82896	1.13E-08	2.29E-08
DNAH17-AS1	1.572	-0.34034	1.18E-08	2.40E-08
LINC00624	1.505774	-0.02876	1.20E-08	2.44E-08
LINC01805	2.642002	-2.74854	1.35E-08	2.74E-08
LINC01271	1.473607	-1.50957	1.40E-08	2.82E-08
ARLNC1	2.040939	-1.61286	1.41E-08	2.84E-08
LINC00973	3.681587	-1.71993	1.43E-08	2.89E-08
LINC02601	1.333341	-2.76319	1.53E-08	3.07E-08
LINC01482	-1.02437	-2.11428	1.61E-08	3.23E-08
TTLL11-IT1	2.017878	-2.87211	1.92E-08	3.84E-08
CASC18	1.712374	-1.23602	2.13E-08	4.24E-08
MIR3681HG	2.153973	-1.86076	2.14E-08	4.26E-08
BANCR	-1.71433	-0.70669	2.14E-08	4.28E-08
TTLL10-AS1	-1.37224	-1.49995	2.19E-08	4.36E-08
LINC02418	5.740519	-0.96351	2.28E-08	4.53E-08
LINC02152	4.189557	-2.593	2.35E-08	4.67E-08
MIR3150BHG	1.338045	-2.0652	2.37E-08	4.72E-08
LINC02580	-1.14873	-1.96797	2.50E-08	4.96E-08
LINC01301	1.086351	-0.28425	2.86E-08	5.65E-08
LINC02327	5.177352	-1.81012	3.23E-08	6.38E-08
DSCR4	5.721046	-2.5317	3.25E-08	6.40E-08
GNAS-AS1	1.553577	-1.48317	3.41E-08	6.71E-08
LINC00092	-1.20227	-0.00517	3.70E-08	7.26E-08

LINC00161	1.893023	-2.7278	4.28E-08	8.36E-08
LINC00621	1.724429	-2.38106	4.33E-08	8.46E-08
LINC02099	1.271021	-1.85636	4.50E-08	8.77E-08
C20orf197	1.890158	0.147329	4.71E-08	9.16E-08
LINC00954	1.339803	0.319286	5.04E-08	9.79E-08
LINC02241	3.911122	-0.24999	5.37E-08	1.04E-07
LINC01446	4.299095	-0.29456	6.23E-08	1.20E-07
ST8SIA6-AS1	3.197774	0.236355	6.33E-08	1.22E-07
LINC01695	1.882409	-2.79934	6.47E-08	1.25E-07
LINC01932	1.420977	-0.40022	6.51E-08	1.25E-07
LINC01602	3.660483	-0.77916	7.58E-08	1.46E-07
ERVH48-1	2.913085	0.559062	8.19E-08	1.57E-07
HAR1A	-1.01143	-1.14109	8.32E-08	1.59E-07
HCP5B	-1.28433	-2.60334	8.71E-08	1.66E-07
LINC00303	3.200796	-2.42411	9.28E-08	1.77E-07
FZD10-DT	1.430982	3.15454	1.08E-07	2.04E-07
ZNF793-AS1	1.234841	0.425925	1.11E-07	2.11E-07
LINC01033	2.771583	-1.8113	1.25E-07	2.37E-07
LINC01694	1.72748	1.610901	1.28E-07	2.41E-07
LINC02188	2.227286	-0.19851	1.28E-07	2.42E-07
LINC00839	1.408774	2.913453	1.32E-07	2.50E-07
LINC00221	5.592535	0.920933	1.37E-07	2.59E-07
IGF2-AS	2.556636	-1.93174	1.43E-07	2.69E-07
LINC00637	1.376	-2.54972	1.66E-07	3.11E-07
TSPEAR-AS2	1.948013	1.597948	1.67E-07	3.14E-07
STPG3-AS1	1.341944	-1.65128	1.84E-07	3.45E-07
LINC01268	-1.22618	-0.09051	2.12E-07	3.96E-07
KRT7-AS	-1.39992	1.411462	2.15E-07	4.00E-07
LINC01354	-1.12282	-0.94371	2.57E-07	4.77E-07
RFPL1S	1.672428	-0.05615	2.62E-07	4.85E-07
LINC00284	-1.44767	-1.94651	2.84E-07	5.25E-07
NALCN-AS1	-1.23828	-1.71114	2.94E-07	5.43E-07
GPR1-AS	3.952135	-2.19352	3.15E-07	5.81E-07
TDRKH-AS1	1.092221	-0.78974	3.25E-07	5.98E-07
GATA3-AS1	2.325055	-2.55575	3.25E-07	5.98E-07
LINC01270	1.136129	0.22936	3.39E-07	6.22E-07
LINC00479	2.055545	-1.5454	3.40E-07	6.25E-07
C11orf72	1.266074	-2.68702	3.67E-07	6.73E-07
SYNPR-AS1	1.315788	-0.86559	3.99E-07	7.29E-07
LINC00189	-1.14992	-1.86728	4.25E-07	7.77E-07
LINC02576	1.297001	-0.74812	4.51E-07	8.22E-07
LINC00534	2.300226	-2.97633	4.65E-07	8.47E-07
ACTN1-AS1	1.068713	-2.81684	5.04E-07	9.15E-07
LINC00880	1.418962	-2.47169	5.35E-07	9.71E-07
LINC01485	1.56477	-2.26403	5.79E-07	1.05E-06
PCAT18	3.197476	-1.71517	5.84E-07	1.06E-06
C12orf77	3.113687	-2.71503	6.23E-07	1.12E-06
LINC01281	1.42039	-2.12138	6.37E-07	1.15E-06
UPK1A-AS1	2.606832	-2.34453	6.74E-07	1.21E-06
LINC01515	1.179107	0.732943	7.26E-07	1.30E-06
DIAPH2-AS1	1.297321	-1.1268	7.87E-07	1.41E-06
CASC15	1.017192	2.894567	7.91E-07	1.42E-06
LINC02487	2.041189	-1.37475	8.11E-07	1.45E-06
LINC01539	1.837346	-2.3385	8.27E-07	1.48E-06
LINC01619	1.068503	0.564101	8.38E-07	1.50E-06
LINC01993	1.400509	-1.53999	8.66E-07	1.54E-06
LINC01228	3.445145	-1.97376	9.04E-07	1.61E-06
TCL6	2.160798	-0.15294	9.89E-07	1.76E-06

LINC02042	2.182315	-2.83523	1.05E-06	1.85E-06
LINC02506	3.309065	-1.73754	1.17E-06	2.07E-06
CELF2-AS1	-1.11371	-1.47528	1.22E-06	2.15E-06
AATBC	1.024581	2.551023	1.23E-06	2.17E-06
ISPD-AS1	1.732195	-2.29835	1.25E-06	2.21E-06
LINC00237	2.913449	-2.64365	1.29E-06	2.27E-06
LINC01537	-1.3443	-1.15195	1.34E-06	2.35E-06
LINC01152	1.832266	-1.08712	1.39E-06	2.44E-06
LINC02450	1.56042	-1.82932	1.52E-06	2.66E-06
LINC00540	2.052125	0.72153	1.52E-06	2.67E-06
LINC02261	2.406028	-2.69306	1.71E-06	2.99E-06
LINC00970	2.229959	-2.52429	1.81E-06	3.16E-06
LINC01998	3.255178	-2.5189	1.82E-06	3.19E-06
EGFR-AS1	1.408116	-0.86345	1.96E-06	3.42E-06
LINC02195	1.320478	-1.37477	1.99E-06	3.47E-06
LNCSRLR	1.094428	-2.38187	2.19E-06	3.80E-06
LINC00867	2.560544	-3.04837	2.19E-06	3.81E-06
TTTY20	5.907659	-2.69841	2.25E-06	3.91E-06
OVAAL	2.834165	-2.36901	2.29E-06	3.98E-06
ZNF503-AS1	-1.10338	0.739242	2.30E-06	3.99E-06
FLJ22447	1.301722	0.589547	2.32E-06	4.03E-06
MIR7-3HG	3.767159	-2.73496	2.40E-06	4.16E-06
NALT1	1.118372	-0.28003	3.11E-06	5.36E-06
LINC02575	2.26446	1.431323	3.17E-06	5.46E-06
LINC00939	2.144389	-0.9618	3.61E-06	6.18E-06
SOX1-OT	4.237487	-2.55065	4.06E-06	6.94E-06
DPP10-AS1	2.501168	-0.28795	4.34E-06	7.40E-06
LINC01725	1.05773	-1.40449	4.39E-06	7.48E-06
BASP1-AS1	1.431235	-2.36654	4.65E-06	7.91E-06
LINC01198	2.935316	-3.10755	5.26E-06	8.92E-06
LINC01447	-1.64157	-2.55101	5.41E-06	9.17E-06
LINC01346	4.645144	-2.2587	5.84E-06	9.87E-06
AQP4-AS1	-1.19042	-0.85469	6.10E-06	1.03E-05
LUCAT1	1.315773	1.138086	6.53E-06	1.10E-05
LLPH-DT	1.014711	-1.41637	6.57E-06	1.11E-05
RDH10-AS1	1.08728	0.314575	6.62E-06	1.11E-05
LINC01819	2.579383	-0.28448	6.84E-06	1.15E-05
LINC01579	1.570853	0.267385	8.08E-06	1.35E-05
LINC00543	1.672838	-1.29559	8.50E-06	1.42E-05
LINC00605	1.889583	-0.74624	9.39E-06	1.56E-05
PROX1-AS1	1.809384	-0.54746	9.61E-06	1.60E-05
LINC01029	4.635191	-2.64734	9.89E-06	1.65E-05
WDR11-AS1	-1.01678	-1.86848	1.02E-05	1.69E-05
LINC01634	1.7687	-2.34813	1.09E-05	1.80E-05
LINC01122	1.486815	-1.46117	1.11E-05	1.85E-05
VLDLR-AS1	1.101871	0.408818	1.13E-05	1.88E-05
LINC02577	1.419797	1.239543	1.16E-05	1.92E-05
PEX5L-AS1	5.346102	-1.39477	1.19E-05	1.97E-05
LINC01989	2.64644	-2.75352	1.24E-05	2.05E-05
LINC02036	1.121757	-0.58888	1.74E-05	2.85E-05
FAM87A	1.180904	-1.05179	1.74E-05	2.85E-05
LINC01320	3.071264	-1.78059	1.87E-05	3.05E-05
SIX3-AS1	2.346435	-2.96092	1.90E-05	3.09E-05
LINC00960	1.138058	1.137017	2.14E-05	3.48E-05
EGOT	1.375068	-0.99134	2.25E-05	3.66E-05
MIR4432HG	1.581871	-2.68873	2.31E-05	3.75E-05
BSN-DT	1.271632	-2.68767	2.35E-05	3.80E-05
LINC02228	1.964429	-2.87978	2.60E-05	4.20E-05

LINC02518	1.591678	-1.79603	2.76E-05	4.45E-05
LINC01983	1.646409	-2.46038	3.14E-05	5.04E-05
LINC02028	1.083292	-1.05766	3.38E-05	5.42E-05
THRA1/BTR	2.97167	-2.76334	3.39E-05	5.42E-05
EMX2OS	2.229694	-0.75887	3.60E-05	5.75E-05
LINC01010	1.420393	1.293174	3.63E-05	5.79E-05
LINC00884	1.108935	0.740607	3.72E-05	5.94E-05
LINC01771	1.117684	-2.79803	4.10E-05	6.52E-05
LINC01978	1.163558	-2.72422	4.22E-05	6.71E-05
LINC02572	1.006157	-2.79892	4.36E-05	6.92E-05
TUSC7	3.612592	-2.10368	4.39E-05	6.97E-05
SNAP25-AS1	1.503439	-1.36914	4.44E-05	7.04E-05
LINC02232	2.497525	-2.30835	4.47E-05	7.09E-05
AADACL2-AS1	1.475432	-1.17545	5.09E-05	8.03E-05
DGCR10	1.101723	-2.80993	5.11E-05	8.07E-05
LINC02434	3.654322	-2.85422	5.15E-05	8.13E-05
FLG-AS1	1.147008	1.080413	5.24E-05	8.25E-05
CLDN10-AS1	2.717378	-2.80925	5.37E-05	8.45E-05
LINC01324	3.243799	-2.92111	5.42E-05	8.52E-05
LINC01781	-1.00958	-1.41282	8.01E-05	0.000125
NECTIN3-AS1	1.197793	-1.63867	8.29E-05	0.000129
LCMT1-AS2	1.467485	-1.32367	8.39E-05	0.00013
SLC8A1-AS1	1.024439	-1.78335	8.65E-05	0.000134
TUSC8	2.598657	-2.30844	0.000124	0.000191
KCNK15-AS1	1.065241	-1.28642	0.000134	0.000205
PCAT14	-1.49912	-2.94816	0.000139	0.000213
LINC01169	-1.23321	-1.91763	0.000141	0.000215
LINC02055	2.371302	-1.25095	0.000145	0.000222
PROSER2-AS1	1.083982	-0.52857	0.000161	0.000245
BVES-AS1	1.228139	-1.35018	0.000164	0.00025
LINC01060	1.423254	-2.1011	0.00018	0.000273
LINC00944	1.086757	-0.59986	0.000187	0.000283
LINC01606	2.435069	-1.28798	0.000187	0.000284
PURPL	2.169723	-1.59556	0.000192	0.000291
MIR4527HG	3.00146	-2.38581	0.000196	0.000296
LINC02212	3.187594	-1.93949	0.000205	0.00031
LINC01018	1.10998	1.384852	0.000262	0.000393
PLAC4	1.54295	-0.08585	0.000321	0.000477
LINC02528	2.470396	-2.85449	0.000321	0.000478
LINC01163	1.602454	-2.65417	0.000325	0.000483
SOX9-AS1	1.008869	-0.10649	0.000326	0.000484
LINC01484	1.108902	-0.45386	0.00033	0.00049
LINC01563	1.771041	-2.85176	0.000344	0.00051
JAKMIP2-AS1	1.640716	-1.79199	0.000388	0.000572
LINC00200	3.212781	-2.18519	0.000567	0.000826
TSPEAR-AS1	1.177032	1.141425	0.000604	0.000878
EFCAB6-AS1	1.03986	-2.79806	0.000675	0.000977
DLGAP1-AS5	1.772937	-1.89987	0.000786	0.001132
LINC01413	2.81493	-3.16968	0.000811	0.001167
DRAIC	1.335601	0.552032	0.001061	0.001513
LINC00589	1.077149	-2.32581	0.001088	0.001549
OSTN-AS1	1.299752	-2.48102	0.001169	0.00166
DSCAM-AS1	2.567615	-2.90089	0.001344	0.0019
LINC01293	1.12276	-1.1496	0.001543	0.002172
ALDH1L1-AS2	1.183876	-2.34981	0.001606	0.002259
LINC01502	1.694883	-1.91899	0.002305	0.003202
LINC00703	2.422195	-3.01094	0.002583	0.003571
LINC01508	1.032284	0.869594	0.002753	0.003797

TRHDE-AS1	-1.14808	-0.59973	0.002978	0.004096
LINC02474	1.575443	-2.38563	0.004466	0.006042
LINC02211	1.076768	-2.72773	0.004993	0.006727
LINC02388	1.515383	-3.08049	0.005363	0.007201
LINC00824	1.355121	-2.57474	0.005507	0.007389
SACS-AS1	1.51365	-2.95092	0.008465	0.011155
LINC01785	-1.4814	-2.81554	0.009932	0.012986
HAND2-AS1	1.378396	-1.81076	0.010065	0.013145
KIF25-AS1	1.168936	-0.86981	0.010511	0.013704
MEG8	1.039995	-2.31903	0.011525	0.014975
LINC01820	1.723569	-2.93947	0.012474	0.016161
KCCAT333	1.039341	-0.10406	0.01331	0.017208
LINC00452	1.159632	-2.45783	0.017884	0.022849
LINC01019	1.639824	-2.81217	0.018447	0.023553
LINC02432	1.014724	0.522613	0.021132	0.026839
LINC00603	1.595806	-2.81655	0.026704	0.03359
